# Decoding multicomponent crosstalk: integrated pharmacodynamic‒pharmacokinetic network of sour jujube seed

**DOI:** 10.1186/s13020-025-01265-0

**Published:** 2025-11-20

**Authors:** Yansheng Wu, Lijun Sun, Yimian Ma, Lina Wu, Weitao Niu, Jianyuan Li, Qingmei Feng, Senghu Wang, Hanxue Niu, Qing Bai, Junxia Du, Hualiang Liu

**Affiliations:** 1 Hebei Xingzaoren Utilization Technology Innovation Center, Xingtai University, Xingtai, 054001 China; 2 All China Federation of Supply and Marketing Cooperatives Nanjing Institute for Comprehensive Utilization of Wild Plants, Nanjing, 211111 China; 3 Key Lab of Chinese Medicine Resources Conservation, State Administration of Traditional Chinese Medicine of China, Institute of Medicinal Plant Development, Chinese Academy of Medical Sciences & Peking Union Medical College, Beijing, 100193 China; 4 Hebei Jingxin Agricultural Technology Development Co., Ltd, Xingtai, 054001 China

**Keywords:** Sour jujube seed, Multicomponent, Pharmacodynamic–pharmacokinetic network, Chinese medicinal plant

## Abstract

*Ziziphus jujuba* var. *spinosa* seed (SJS) is a multicomponent traditional remedy with diverse pharmacological activities, including sedative, neuroprotective, anxiolytic, and cardiometabolic effects. However, identifying the bioactive constituents responsible for these broad therapeutic effects and understanding how they interact at the molecular level remains challenging due to the synergistic interactions among numerous phytochemicals. This review introduces an integrated pharmacodynamic–pharmacokinetic (PD–PK) network analysis to systematically decode the compound–target–pathway relationships of SJS. Pharmacokinetic profiling of key constituents (e.g., spinosin, jujuboside A and B) was combined with network pharmacology to map absorbed compounds to their biological targets and pathways. This approach revealed a holistic compound–target network, demonstrating that SJS’s therapeutic efficacy arises from the synergistic effects of multiple constituents. These interacting compounds simultaneously modulate various targets and pathways, including neurotransmitter systems, inflammatory and antioxidant responses, and metabolic regulators. By incorporating PK constraints, non-bioavailable compounds were excluded, isolating core bioactive constituents and linking them to pharmacological actions within a rigorous, systems-level framework. The findings provide a comprehensive understanding of SJS’s multifaceted mechanism of action, addressing previous knowledge gaps and highlighting how the integrated PD–PK paradigm can guide the rational development and clinical translation of SJS-based therapies.

## Introduction

### Therapeutic profile and mechanistic knowledge gap of sour jujube seed (SJS)

SJS is a widely used traditional Chinese remedy for treating insomnia, anxiety, and other neurofunctional disorders [1–10]. Modern pharmacological studies indicate that SJS exerts sedative and anxiolytic effects primarily by modulating γ-aminobutyric acidergic (GABAergic) and serotonergic neurotransmitter systems [11]. Key bioactive constituents of SJS, including saponins (e.g., jujubosides) and flavonoids such as spinosin, have been identified as potential contributors to these therapeutic effects [1, 11]. However, the precise mechanisms by which SJS’s diverse bioactive components mediate its therapeutic outcomes remain poorly understood. Specifically, it is unclear which individual components or their combinations are responsible for the clinical efficacy of SJS and how they interact with biological targets to alleviate disease conditions. This gap in knowledge presents a significant challenge to advancing SJS research and its evidence-based application.

### Research challenges: composition complexity and methodological limitations

One major reason for this knowledge gap is the complexity of SJS’s multicomponent composition and the limitations of conventional research approaches in capturing that complexity. Although SJS contains a wide range of compounds, not all are pharmacologically relevant in vivo due to factors such as poor absorption, low bioavailability, or minimal presence in the raw herb [12]. Traditional reductionist studies that isolate individual ingredients or focus on broad pharmacological effects often fail to reveal the component-effect relationships within such a complex mixture. Even emerging in silico “network pharmacology” analyses, which predict multiple target interactions for various components, have limitations. These methods often fail to account for whether a compound can be absorbed and reach effective concentrations in systemic circulation, leading to the inclusion of compounds that may be pharmacokinetically irrelevant and resulting in unrealistic or oversimplified mechanistic conclusions [13]. In summary, current research strategies struggle to address the multi-component, multi-target nature of SJS, making it difficult to pinpoint which bioactive ingredients drive its therapeutic effects and the biological pathways involved, particularly under physiological conditions.

### PD–PK network analysis: an integrative framework to decipher SJS’s mechanisms

Pharmacodynamic-pharmacokinetic (PD–PK) network analysis has recently emerged as a promising framework to overcome these challenges. This approach integrates classical pharmacokinetic profiling, which tracks a compound’s absorption, distribution, metabolism, and excretion (ADME), with network-based pharmacodynamic analysis to examine the compound’s biological targets and pathways. A PD–PK network analysis of SJS involves identifying its numerous constituents and determining which of these reach meaningful levels in the blood or target tissues after administration (the PK aspect). It then maps those absorbed compounds onto a network of protein targets, pathways, and disease processes they influence (the PD aspect). By combining these dimensions, this approach systematically elucidates the complex relationships between SJS’s chemical constituents and their therapeutic mechanisms. Notably, integrating pharmacokinetic data filters out components that do not reach systemic circulation, focusing the analysis on a subset of bioavailable compounds. Network pharmacology then connects these compounds to potential targets and signaling pathways, providing a holistic view of how SJS exerts its effects at the systems level. Recent studies underscore the power of this integrated strategy. For example, one investigation identified 12 phytochemicals in a traditional Chinese medicine (TCM) decoction, but only five were detected in plasma based on pharmacokinetic evaluation. Network analysis further narrowed down four key compounds as the likely “material basis” of the formula’s efficacy [12]. These findings demonstrate how PD–PK network analysis can distinguish core active constituents from background noise and link them to pharmacological actions in a data-driven, rigorous manner.

### Advancing SJS research and application through a PD–PK network paradigm

By identifying which components of SJS are both bioavailable and biologically potent, the PD–PK network paradigm addresses critical knowledge gaps left by previous approaches. This framework enables researchers to systematically connect SJS’s chemical makeup to its disease-modifying effects through integrated component–target–pathway analysis, coupled with quantitative pharmacokinetic validation. In doing so, it accounts for synergistic multi-component interactions while adhering to fundamental pharmacological principles, such as exposure–response relationships. Ultimately, adopting a PD–PK network analysis approach provides a more robust and predictive understanding of SJS’s therapeutic profile. It offers a cohesive strategy for unraveling how SJS’s core bioactive constituents interact with neurobiological networks and how these interactions translate into clinical benefits. This improved conceptual foundation is expected to advance the scientific understanding of SJS’s mechanisms of action and guide more effective utilization and further development of SJS-based therapies. In the following sections of this review, we expand on this framework to examine current evidence and highlight how an integrated PD–PK network perspective can drive forward research and application of sour jujube seed in modern medicine.

## Pharmacodynamics

**Fig. 1:**
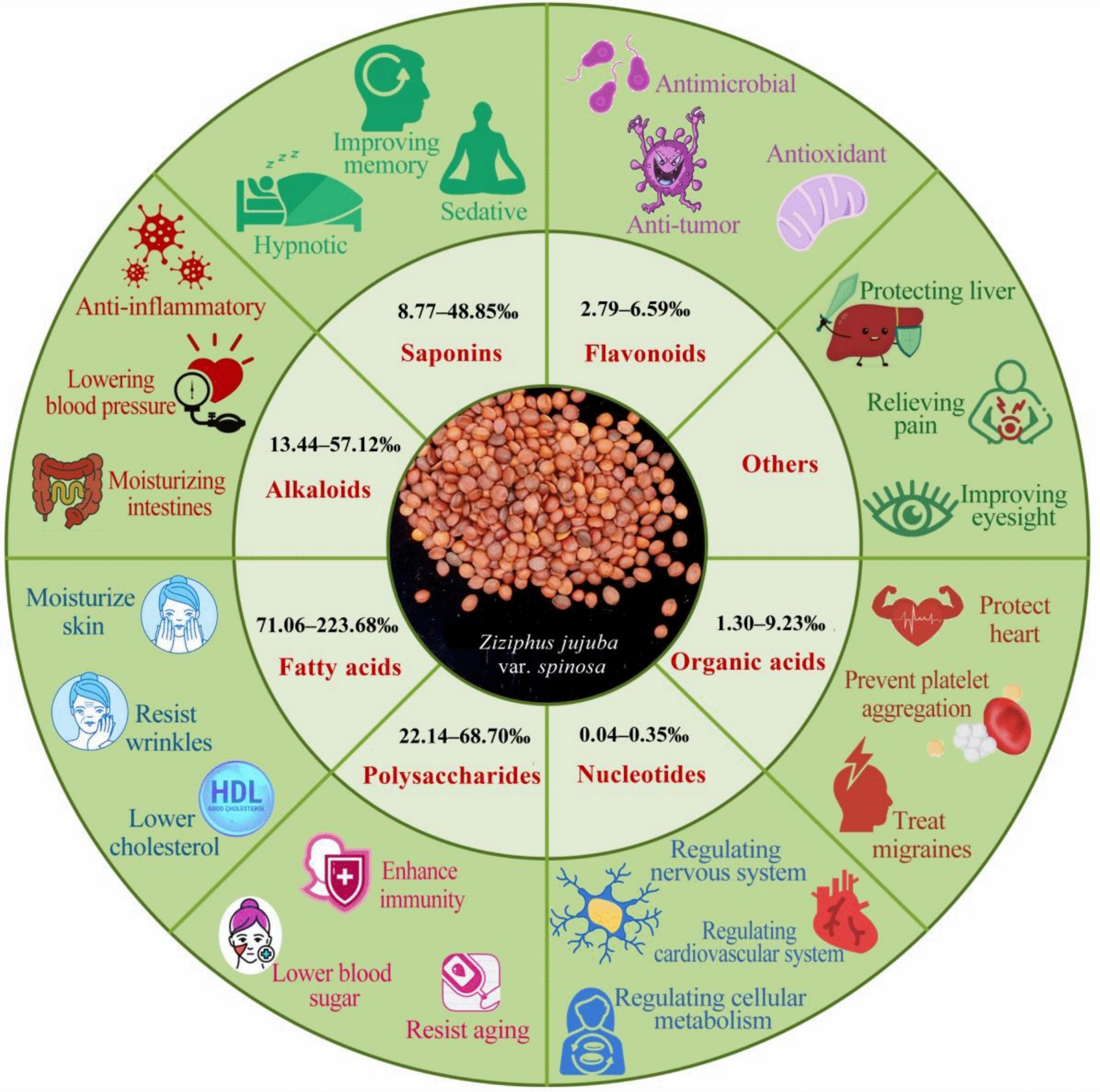
Main chemical compounds and pharmacodynamic effects of SJS

Advances in natural product research have brought SJS to the forefront due to its broad pharmacodynamic activities. Bioactivity-guided fractionation has identified key bioactive constituents in SJS, including flavonoids (such as spinosin, vitexin-II, swertisin, 6‴-feruloylspinosin, 6‴-*p*-coumaroylspinosin, and kaempferol-3-O-rutinoside), saponins (comprising both tetracyclic and pentacyclic triterpenoid subclasses), alkaloids (pyridine, isoquinoline, cyclopeptide, and aporphine types), and oils rich in unsaturated fatty acids (oleic acid, linoleic acid, linolenic acid) as well as saturated fatty acids (palmitic acid, stearic acid), primarily esterified in triglycerides (Fig. 1).

**Fig. 2:**
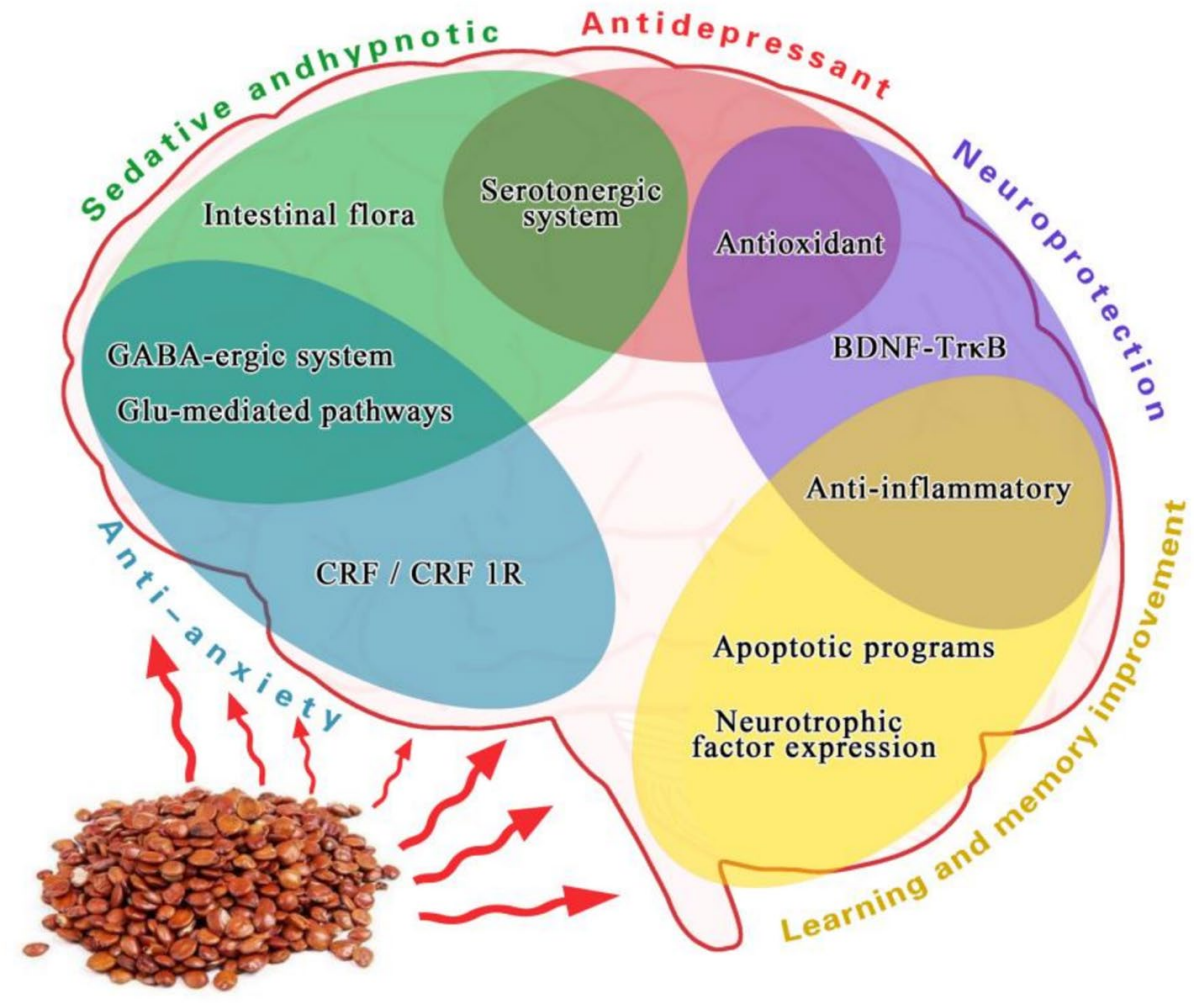
SJS-associated central nervous system (CNS) alterations and mechanisms

These constituents contribute to the multifaceted therapeutic effects of SJS, including antioxidant, sedative-hypnotic, neuroprotective, antidepressant, anxiolytic, antitumor, immune-enhancing, and cardiovascular-regulating activities, as well as improvements in learning and memory functions (Fig. 2). These effects are primarily mediated through modulation of GABAergic neurotransmission, activation of serotonergic pathways, and restoration of redox homeostasis.

### Sedative and hypnotic effects

#### Distinction between sedation and hypnosis: mechanisms and clinical applications

Sedation and hypnosis are both pharmacologically induced states of central nervous system (CNS) depression, but they differ in their depth and effects. Sedation promotes relaxation, drowsiness, and reduced anxiety without causing sleep, primarily through GABAergic activity. It is commonly used for anxiety management, pre-surgical sedation, and muscle relaxation. In contrast, hypnosis induces a deeper, sleep-like state characterized by reduced responsiveness and increased suggestibility. Hypnotic drugs, such as propofol, also affect serotonin and melatonin, altering sleep architecture and brainwave patterns (e.g., REM and theta waves). While both states share GABAergic mechanisms, hypnosis also involves modulation of excitatory neurotransmitters like glutamate. Higher doses of sedatives can induce hypnosis-like states, blurring the distinction. Sedation is typically used for milder conditions, while hypnosis is applied for deeper sleep or therapeutic purposes requiring heightened suggestibility.

#### SJS as a multimechanistic alternative to Western sleep medications

Both sedation and hypnosis play critical roles in maintaining physiological rhythms, regulated by complex networks involving neurotransmitter systems, gaseous signaling molecules, and the microbiota–gut–brain axis [14, 15]. Although Western medications demonstrate significant efficacy in improving sleep quality, prolonged use may lead to dependency. Additionally, the adverse effects associated with Western pharmacological treatments for sleep disorders are often more pronounced compared to those of traditional Chinese medicine [16]. SJS, whether used alone or in combination with other herbs, is widely recognized for its sedative and hypnotic properties. Clinically, SJS is an effective treatment for insomnia and a means of mitigating dependence on Western medications [17]. The sedative-hypnotic effects of SJS are mediated through a sophisticated multireceptor pharmacodynamic mechanism involving GABAergic, serotonergic, and circadian pathways, with distinct bioactive constituents operating synergistically.

#### Saponins: core bioactives for mitigating excitotoxicity and regulating sleep architecture

**Fig. 3:**
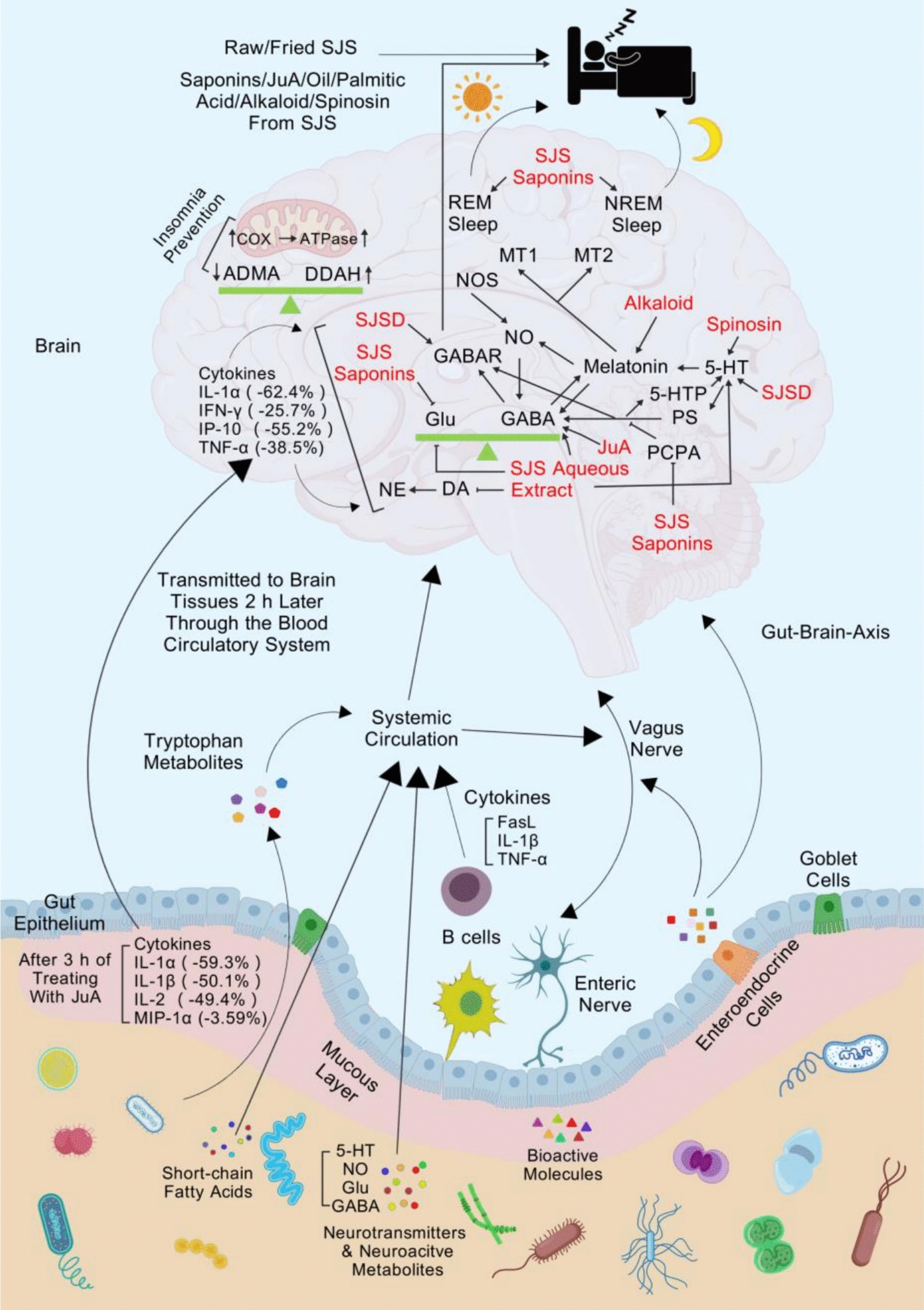
SJS bioactive compounds’ sedative-hypnotic mechanism through gut–brain axis. Created with BioGDP.com [22]

Total saponins derived from SJS can mitigate excitatory amino acid toxicity in the brains of elderly rats with anemia-induced insomnia and alleviate neuronal injury related to glutamate (Glu) and GABA [18]. Saponins have been shown to outperform flavonoids in sedative and hypnotic activity, while polysaccharides do not exhibit these effects [19]. The chronobiological efficacy of jujubosides manifests as photoperiod-synchronized somnolence in rodent models, which is primarily regulated through serotonergic receptor trafficking in the suprachiasmatic nucleus-pineal axis [20]. Jujubosides also extend total and rapid eye movement (REM) sleep and increase non-rapid eye movement (NREM) sleep duration, particularly light sleep. Furthermore, jujubosides enhance the hypnotic effect of sodium pentobarbital, with additional potentiation by 5-hydroxytryptophan (Fig. 3). Jujubosides also counteract the hypnotic inhibitory effects induced by para-chlorophenylalanine on sodium pentobarbital. Jujubosides combat insomnia pathogenesis by accelerating the catabolism of asymmetric dimethylarginine (ADMA) through upregulation of dimethylarginine dimethylaminohydrolase (DDAH) in sleep-restricted rodents [21].

#### Jujubosides A (JuA) and sleep–wake regulation: from molecular targets to network effects

**Fig. 4:**
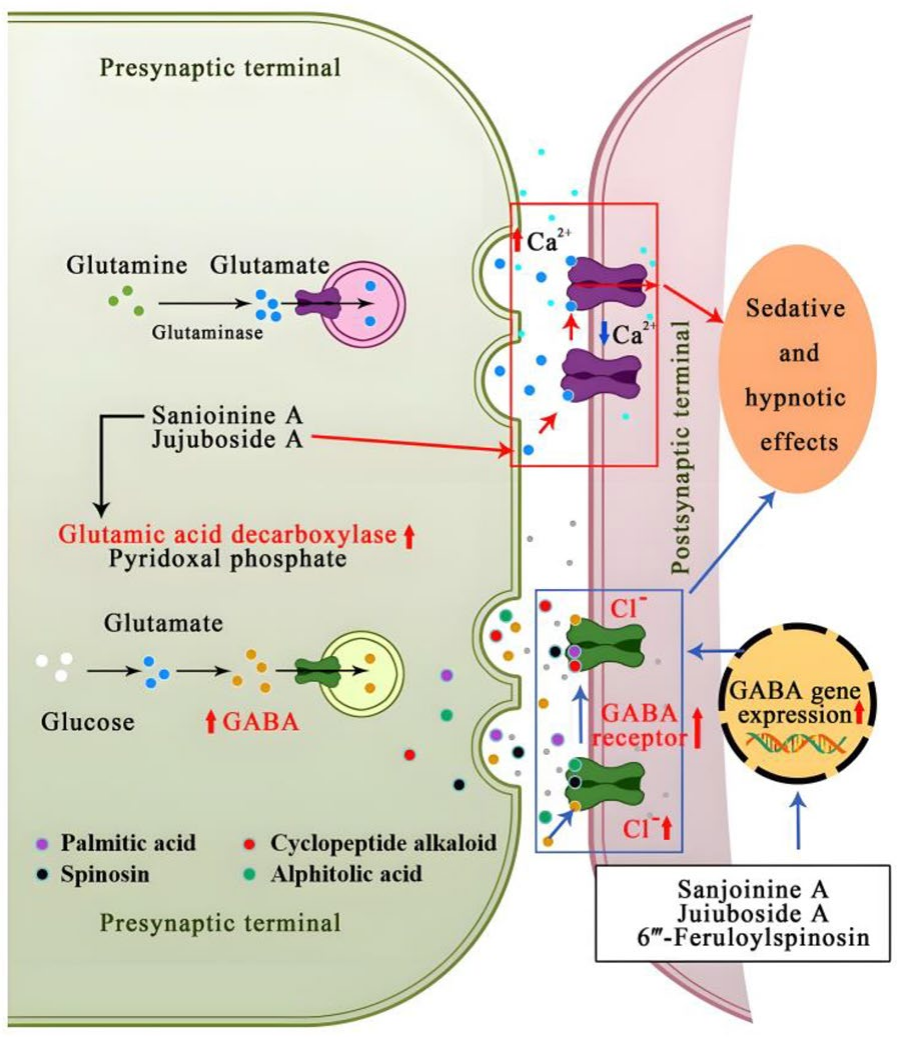
SJS compounds mediate sedative-hypnotic effects via GABA/glutamate synaptic regulation in presynaptic and postsynaptic terminals

Research indicates that JuA ameliorates insomnia by promoting cyclooxygenase (COX) expression in cardiac mitochondria and restoring adenosine triphosphatase (ATPase) activity via membrane potential modulation [4]. JuA-mediated remodeling drives differential expression of hippocampal GABA type A (GABAA) receptor α1/α5/β2 subunit genes [23]. The GABAA α1 subunit is primarily involved in sedative and hypnotic effects, while the GABAA α5 subunit mediates muscle relaxation, accelerating the onset of sedation and hypnosis. JuA achieves its hypnotic–sedative specificity by regulating GABAA receptor gene expression (Fig. 4) and attenuating gut-derived inflammatory cytokine signaling to neuronal circuits, paralleling melatonin’s mechanism (Fig. 3) [24]. Both in vivo and in vitro, JuA inhibits rat hippocampal formation activity, reducing the excitatory postsynaptic potential (EPSP) slope by blocking glutamate-mediated excitation and antagonizing calmodulin. High-dose JuA suppresses hippocampal *Cornu Ammonis* 1 (CA1) hyperactivity induced by sodium penicillin [25], with stronger effects on the amplitude and latency of the first population spike compared to the inhibition of the EPSP slope. JuA significantly reduces EPSP slope and population spike amplitude in granule cell responses, similar to its effects on CA1 pyramidal cell responses [26]. Additionally, JuA acts as a noncompetitive inhibitor of calmodulin (CaM) and has inhibitory effects on mouse activity [27]. Notably, JuA does not induce sleep directly but enhances sodium pentobarbital-induced sleep behavior by modifying the GABAergic system [28]. Some scholars suggest that the true agents responsible for sedative and hypnotic effects may not be the saponins themselves but their metabolites. Specifically, jujuboside B (JuB) and jujubogenin, hydrolysis products of primary saponins, may be the absorbed bioactive species responsible for sedative effects via GABAA receptor modulation [29].

#### Flavonoids: modulating CNS activity and enhancing sleep via serotonergic and GABAergic pathways

Total flavonoids from SJS inhibit spontaneous activities in experimental mice, prolong sleep duration, and significantly suppress CNS excitation. The sedative-enhancing effect of spinosin, a C-glycosyl flavone in SJS, on sodium pentobarbital-induced sleep involves serotonergic pathways [30]. The antagonism of postsynaptic 5-hydroxytryptamine-1A (5-HT1A) receptors may underlie spinosin’s selective REM sleep enhancement in pentobarbital-treated rats, alongside its overall sleep-prolonging and latency-reducing effects [31]. Both spinosin and 6‴-feruloylspinosin converge on GABAergic targets, co-upregulating hippocampal GABAAα1/α5 and GABAB receptor 1 transcripts, thereby potentiating GABA receptor activity as the primary sedative mechanism.

#### SJS oils: identification as the potent hypnotic fraction and dosage considerations

Different extraction methods exhibit similar effects in inhibiting spontaneous activities, accelerating sleep onset, extending total sleep time, and consolidating sleep bouts in mice. SJS oil-induced inhibition of spontaneous activities increases with prolonged administration without tolerance development. SJS oil represents the active constituent responsible for the tranquilizing and hypnogenic potency of SJS. Crude drug dosage calculations indicate that the effective dosage range for SJS oil is 10–20 g, whereas the dosage for jujubosides is hundreds of times greater, confirming the oil as the active component. High-performance liquid chromatography (HPLC) analysis of raw and fried SJS, utilizing a stepwise gradient elution strategy, reveals no differences in chemical component types but variations in the content of certain chemicals. Both raw and fried SJS exhibit significant sedative and hypnotic effects, with no notable pharmacodynamic disparities at equivalent doses, permitting the clinical use of both [32]. SJS oil enhances sleep onset rates in mice at subthreshold sodium pentobarbital doses and prolongs sleep duration at suprathreshold doses. Receptor-ligand binding studies identify palmitic acid (a saturated fatty acid), alphitolic acid (a lupane-type triterpenoid), and spinosin as the bioactive constituents responsible for the observed sedative and hypnotic effects of SJS [33].

#### Alkaloids: contributions to sedative effects through GABA receptor modulation

Following intragastric administration of SJS alkaloids to mice, spontaneous activities gradually decrease, and sleep quality and duration improve significantly [34]. The cyclopeptide alkaloid fraction of SJS may play a pivotal role in potentiating sodium pentobarbital-induced sleep behavior by enhancing chloride ion influx at the GABA receptor [35].

#### Integrated analysis identifies core insomnia-targeting components in SJS

Sanjoinine, spinosin, apigenin, JuA, and higenamine have been identified as the core active components responsible for SJS’s efficacy against insomnia based on integrated serochemical profiling and pharmacodynamic network analysis. Triterpenoid saponins exert sedative effects via GABAA targets, while alkaloids influence melatonin pathways [36]. In addition to monotherapy, SJS-herb combinations potentiate sedative–hypnotic responses. Notably, SJS decoction (SJSD), a classical Chinese formulation primarily containing SJS, possesses sedative and hypnotic properties [11, 37], with binding affinity for GABA receptors as well as serotonergic receptor subtypes 5-HT1A and 5-HT2 [38]. Convergent findings have been reported for combinations of SJS aqueous extracts with *Salvia miltiorrhiza* ether extracts [39].

#### Polysaccharides: limited role in sedative–hypnotic activities

SJS polysaccharides do not significantly inhibit spontaneous movements or coordinated motor activities in normal mice, nor do they prolong sleep duration in suprathreshold barbital sodium-treated mice or increase sleep episodes in subthreshold barbital sodium-treated mice. Comparative analysis reveals no statistically significant variation relative to the control conditions. Thus, SJS polysaccharides exhibit no or only weak hypnotic effects [40].

#### Summary: the polypharmacodynamic profile and therapeutic advantages of SJS

This comprehensive evidence establishes SJS as a multi-mechanistic sedative–hypnotic agent, offering three key insights. First, its efficacy arises from synergistic interactions between distinct bioactive fractions—oils (most potent), saponins (particularly jujuboside A and B metabolites), flavonoids (spinosin), and alkaloids—each engaging complementary pathways: GABAA receptor modulation (subunit-specific gene regulation, Cl⁻ influx), serotonergic (5-HT1A antagonism), circadian synchronization, and mitochondrial/metabolic regulation. Second, critical differential efficacy exists: oils demonstrate superior potency at clinically relevant doses, while polysaccharides are inactive, highlighting the importance of constituent-specific mechanisms. Third, SJS exhibits polypharmacodynamic sophistication: it enhances endogenous sleep systems (melatonin pathways), suppresses excitotoxicity (Glu/GABA rebalancing, CaM inhibition), and amplifies hypnotic drugs through pharmacokinetic and pharmacodynamic interactions. The multireceptor engagement profile—spanning GABAA, 5-HT1A/2, and circadian regulators—enables broad-spectrum efficacy against diverse insomnia etiologies while maintaining physiological sleep architecture. This mechanistic richness, combined with formulation-dependent optimization in traditional preparations, positions SJS as a uniquely versatile neuropharmacodynamic agent for sleep disorders.

### Neuroprotective effects

#### Multitarget neuroprotection of SJS extracts and jujubosides against neural injury

SJS extracts and their bioactive jujubosides provide robust neuroprotection against various forms of neural injury through multitarget modulation of pathological cascades. The neuroprotective effects of SJS methanol extracts have been demonstrated in N-methyl-D-aspartic acid (NMDA)-induced rat cerebellar granule neuron cultures [41]. Jujubosides protect against cerebral ischemic injury by reducing malondialdehyde (MDA) levels in the cerebrum of ischemia model rats, while also increasing the activities of superoxide dismutase (SOD), creatine kinase, and lactate dehydrogenase. Furthermore, they decrease lactate content and alleviate cerebral neuronal damage. Jujubosides mitigate lipid peroxidation injury induced by cerebral ischemia, likely through enhanced SOD activity. Notably, JuA significantly improves histopathological damage induced by β-amyloid 1–42 (Aβ_1–42_) [42]. Ethanol extracts mediate plasmin activation to improve therapeutic efficacy against Alzheimer’s disease (AD)-like manifestations in 5XFAD models [43]. Aβ-induced deficits in hippocampal long-term potentiation are reversed by SJS treatment through regulation of brain-derived neurotrophic factor (BDNF)/tyrosine kinase receptor B (TrκB) signaling, induced by SJS-mediated post-translational modifications of BDNF [44]. Ethanol extracts also enhance synaptic transmission in the hippocampus by increasing alpha-amino-3-hydroxy-5-methyl-4-isoxazolepropionic acid receptor (AMPAR) transmission via the adenylate cyclase (AC), p21-activated kinase (PAK), and mitogen-activated protein kinase (MAPK) pathways [45].

#### Summary: multi-axis neuroprotection

Collectively, SJS neuroprotection operates through a multi-axis defense strategy: attenuating oxidative stress via SOD upregulation and MDA suppression, preserving metabolic integrity in ischemia, countering Aβ toxicity, and enhancing synaptic plasticity through BDNF/TrkB signaling and AMPAR potentiation. Importantly, its saponins and ethanol-soluble compounds engage both extracellular (plasmin-mediated Aβ clearance) and intracellular (MAPK/AC-PAK synaptic modulation) pathways, while systemically resolving neuroinflammation and redox imbalance. The convergence of these mechanisms—spanning excitotoxicity, proteotoxicity, metabolic crisis, and synaptic failure—positions SJS as a holistic neuroprotectant capable of intercepting neurodegenerative cascades at multiple pathological stages.

### Cognition enhancement

#### SJS and bioactive compounds improve cognitive function in preclinical models

A substantial body of preclinical evidence highlights the therapeutic potential of SJS and its bioactive constituents in improving cognitive function across various pathological models. SJS and *Fructus Gardeniae* extracts enhance performance in learning and memory impairment models in mice, as assessed by the step-down and Morris water maze tests. Composite extracts demonstrate synergistic effects on learning and memory [46]. The aqueous extract of SJS has shown potential in ameliorating alcohol-induced memory retrieval deficits, potentially contributing to enhanced learning ability. Spinosin, jujuboside A, and jujuboside B (JuA/B) are believed to be the primary contributors to this activity [47].

#### SJS constituents ameliorate Alzheimer’s-related cognitive deficits via multi-target mechanisms

In Morris water maze experiments, sevoflurane did not affect learning or memory in mice, though it was potentially neurotoxic. A 10 day intraventricular administration of 0.025 µg µL^−1^ jujubosides improved learning and memory in Alzheimer’s disease (AD) model mice by mitigating sevoflurane-induced mitochondrial-related apoptotic signaling, reducing β-amyloid aggregation, decreasing Tau protein phosphorylation, and inhibiting hippocampal neuronal apoptosis [48]. The cognitive deficits induced by Aβ_1–42_ were significantly reversed by intraventricular JuA administration, as validated by standardized behavioral paradigms [42]. SJS flavonoids prolonged latency, reduced error frequency, and shortened total shock duration, suggesting improved learning and memory in impaired mice. The memory-enhancing effects of spinosin may partially involve the serotonergic neurotransmitter system [49]. Its ability to ameliorate cognitive deficits in Aβ_1–42_-induced AD model mice may also involve modulating a multifaceted pathological axis, including redox imbalance, neuroinflammation, programmed neuronal death, and impaired neurotrophic support [50]. Spinosin mitigates Alzheimer’s-related cognitive dysfunction via polypharmacodynamic modulation of disease-relevant targets. Flavonoid metabolites are also believed to genuinely improve mnemonic processing. The 6‴-*p*-coumaroylspinosin metabolite rescues cognitive deficits via transcriptional upregulation of the glutamate ionotropic receptor kainate type 1 (GluK1), GluK2, and GluK3 subunits in rat hippocampal neurons [51]. Furthermore, these metabolites exhibit notable synergistic effects.

#### Summary: multitarget therapeutic potential of SJS for cognitive disorders

In summary, these findings illustrate the significant potential of SJS and its key components—spinosin, JuA/B, and flavonoid metabolites—as multitarget therapeutic agents for treating cognitive disorders. Their efficacy spans diverse etiologies, including alcohol-induced deficits, anesthetic neurotoxicity, and AD pathology, demonstrably improving performance in standardized behavioral tests. The mechanisms involve sophisticated polypharmacodynamic effects: direct targeting of core AD pathologies (reducing Aβ aggregation and inhibiting Tau hyperphosphorylation); neuroprotection via suppression of mitochondrial apoptosis, oxidative stress, and neuroinflammation; modulation of critical neurotransmitter systems (serotonergic and glutamatergic, the latter potentiated by metabolite-induced GluK receptor upregulation); and enhancement of synaptic plasticity and neuronal survival. Crucially, synergistic effects are evident both within SJS metabolites and in polyherbal formulations. This multipronged approach, which simultaneously addresses amyloidogenesis, tauopathy, excitotoxicity, synaptic dysfunction, and neuronal death, aligns with the complex, multifactorial nature of cognitive decline and neurodegeneration, positioning SJS-derived compounds as promising candidates for developing novel cognitive therapeutics. This mechanistic complexity underscores the need for further exploration of standardized formulations to optimize clinical translatability.

### Anxiolytic effects

#### Anxiolytic mechanisms of SJS: central amygdala modulation and multi-receptor engagement

SJS and its bioactive constituents exhibit significant anxiolytic efficacy through precise neuromodulation in the central amygdala (CeA), targeting key neurochemical systems involved in stress and anxiety responses. In the CeA, SJS aqueous extract alleviates nicotine abstinence anxiety by modulating noradrenergic transmission and corticotropin-releasing factor/corticotropin-releasing factor type 1 receptor (CRF/CRF1R) signaling [52]. Co-regulation of CeA CRF-N/OFQ (neuropeptide nociceptin/orphanin FQ) and its receptor underlies the methanol extract’s ability to mitigate ethanol abstinence symptoms [53]. The anxiolytic effect of ethanol extracts may involve increased GABA and GABAA receptor 1 expression, alongside reduced glutamate (Glu) and NMDA receptor 1 (NMDAR1) expression [1]. Alkaloids and flavonoids in SJS are primarily recognized for their antidepressant effects, and compared to the known anxiolytic diazepam, cyclopeptide alkaloids in SJS may exert similar anxiolytic effects potentially mediated by GABAA receptors [54]. Sanjoinine A, a major alkaloid in SJS, demonstrates comparable anxiolytic responses across elevated plus-maze, hole-board, and open-field challenges, likely through GABAergic transmission [55]. Additionally, spinosin shows anxiolytic activity in preclinical anxiety paradigms, with pharmacodynamic profiling suggesting that modulation of both GABAergic (GABAA) and serotonergic (5-HT1A) receptor systems is the underlying mechanism [56].

#### Summary: multitarget anxiolytic mechanisms of SJS

In summary, SJS exerts anxiolytic effects through multilayered modulation of critical neurocircuitry. Within the CeA, SJS extracts target distinct yet convergent pathways: aqueous extracts reduce substance withdrawal anxiety by normalizing dysregulated noradrenergic transmission and dampening CRF/CRF1R signaling, while ethanol extracts restore excitatory-inhibitory balance by enhancing GABAergic tone (increased GABA/GABAA receptor 1) and suppressing glutamatergic hyperactivity (reduced Glu/NMDAR1). The identification of specific bioactive agents further refines this understanding: cyclopeptide alkaloids and sanjoinine A act primarily through GABAA receptor potentiation, mimicking benzodiazepine-like effects, while the flavonoid spinosin engages both GABAA and 5-HT1A receptors. Additionally, SJS modulates neuropeptide systems, as evidenced by methanol extract-mediated normalization of CeA CRF-N/OFQ co-regulation during ethanol withdrawal. This multitarget engagement—spanning classical neurotransmitters (GABA, Glu, norepinephrine), neuropeptides (CRF, N/OFQ), and their receptors—positions SJS as a unique botanical modulator capable of rectifying multiple nodes within the dysregulated anxiety neurocircuitry. Its efficacy in clinically relevant models of substance withdrawal anxiety further underscores its potential therapeutic value for complex, stress-related psychiatric conditions, where conventional single-target agents often show limited efficacy.

### Antidepressant effects

#### Antidepressant mechanisms of SJS: neurotrophic signaling and bioactive flavonoids

In addition to its anxiolytic effects, SJS exhibits significant neuropharmacodynamic activities relevant to mood regulation, with emerging evidence supporting its antidepressant efficacy through both isolated bioactive compounds and synergistic herbal formulations. JuA may mediate antidepressant effects in murine models by upregulating neurotrophic signaling proteins, including brain-derived neurotrophic factor (BDNF), TrkB, and cAMP-responsive element-binding protein (CREB), while also potentiating hippocampal neuronal activity [57]. High-performance liquid chromatography (HPLC) analysis has identified flavonoid constituents in SJS with anxiolytic and sedative properties, such as vicenin II (apigenin 6,8-di-C-glucoside), the 7-O-glucosyl derivative of spinosin, and two acylated spinosin derivatives: 6‴-feruloylspinosin, 6‴-(4-hydroxybenzoyl) spinosin, along with the parent compound, spinosin itself [58].

#### Synergistic antidepressant effects of SJS-containing herbal formulations

The SJS–*Albizia julibrissin* formula, a TCM prescription for depressive disorders, contains SJS and the stem bark of *Albizia julibrissin*. Its ethanol extract exhibits antidepressant-like effects through mechanisms involving serotonergic, noradrenergic, and monoamine oxidase systems [59]. Additionally, saponin extracts from this formula have been shown to be effective antidepressant components [60], with mechanisms similar to those of ethanol extracts and potential involvement in antioxidant systems. A sour jujube–lily powder suspension also alleviates depressive behaviors in animal models, likely by increasing peripheral serotonin (5-HT) and cerebral 5-hydroxyindoleacetic acid (5-HIAA) levels [61]. SJS enhances exploratory drive and locomotor activation in comorbid anxiety–depression models, independent of *Albizia julibrissin* flower coadministration. Botanical agents and their synergistic formulations modulate monoaminergic neurotransmitter and neurotrophic factor levels in the hippocampus, protecting hippocampal neurons by promoting BDNF production through increased CREB phosphorylation [62]. When compared to single-herb treatments, the combined use of both herbs offers superior anxiolytic and antidepressant effects. The antidepressant mechanism may involve the upregulation of GRP78 (glucose-regulated protein 78), inhibition of caspase-12 expression, mitigation of endoplasmic reticulum stress-induced apoptosis, and stabilization of the endoplasmic reticulum in the hippocampal CA3 region [63]. This herb pair also reduces caspase-12 expression and inhibits apoptosis in the hippocampal CA3 region [64]. Moreover, it ameliorates depressive behaviors in sleep-deprived depression model rats, likely through modulation of 5-HT and 5-HIAA levels in both the hippocampus and serum. Magnoflorine, a major alkaloid in SJS, also exhibits robust antidepressant properties [65].

#### Summary: synergistic formulations and system-level neuromodulation

Overall, these studies outline a multilayered neuropharmacodynamic framework for the antidepressant actions of SJS. The triterpenoid JuA serves as a core neuromodulator, upregulating hippocampal BDNF/TrkB/CREB signaling—a critical pathway for neuroplasticity and mood regulation. The alkaloid magnoflorine independently demonstrates antidepressant activity. Crucially, SJS exerts its most potent neuropsychiatric effects within synergistic formulations, particularly with *Albizia julibrissin*. This herb pair operates through convergent mechanisms: enhancing monoaminergic transmission (serotonergic and noradrenergic systems), inhibiting monoamine oxidase activity, modulating stress-responsive pathways (reducing endoplasmic reticulum stress via GRP78 upregulation and caspase-12 suppression in hippocampal CA3 neurons), and increasing peripheral and central 5-HT/5-HIAA levels. The superior efficacy of combination therapy over monotherapy underscores the importance of system-level network regulation. SJS components simultaneously increase neurotrophic support, mitigate neuronal apoptosis, restore neuroendocrine balance, and counteract oxidative stress. This holistic approach addresses both neurochemical deficits and cellular resilience in depression pathophysiology. These findings position SJS-containing formulations as promising multitarget interventions for mood disorders, capable of modulating interconnected neuroplasticity, stress response, and neurotransmitter systems beyond conventional monoamine reuptake inhibition.

### Antitumor activity

#### Multitargeted antitumor efficacy of jujubosides: from apoptosis to necroptosis across multiple malignancies

**Fig. 5:**
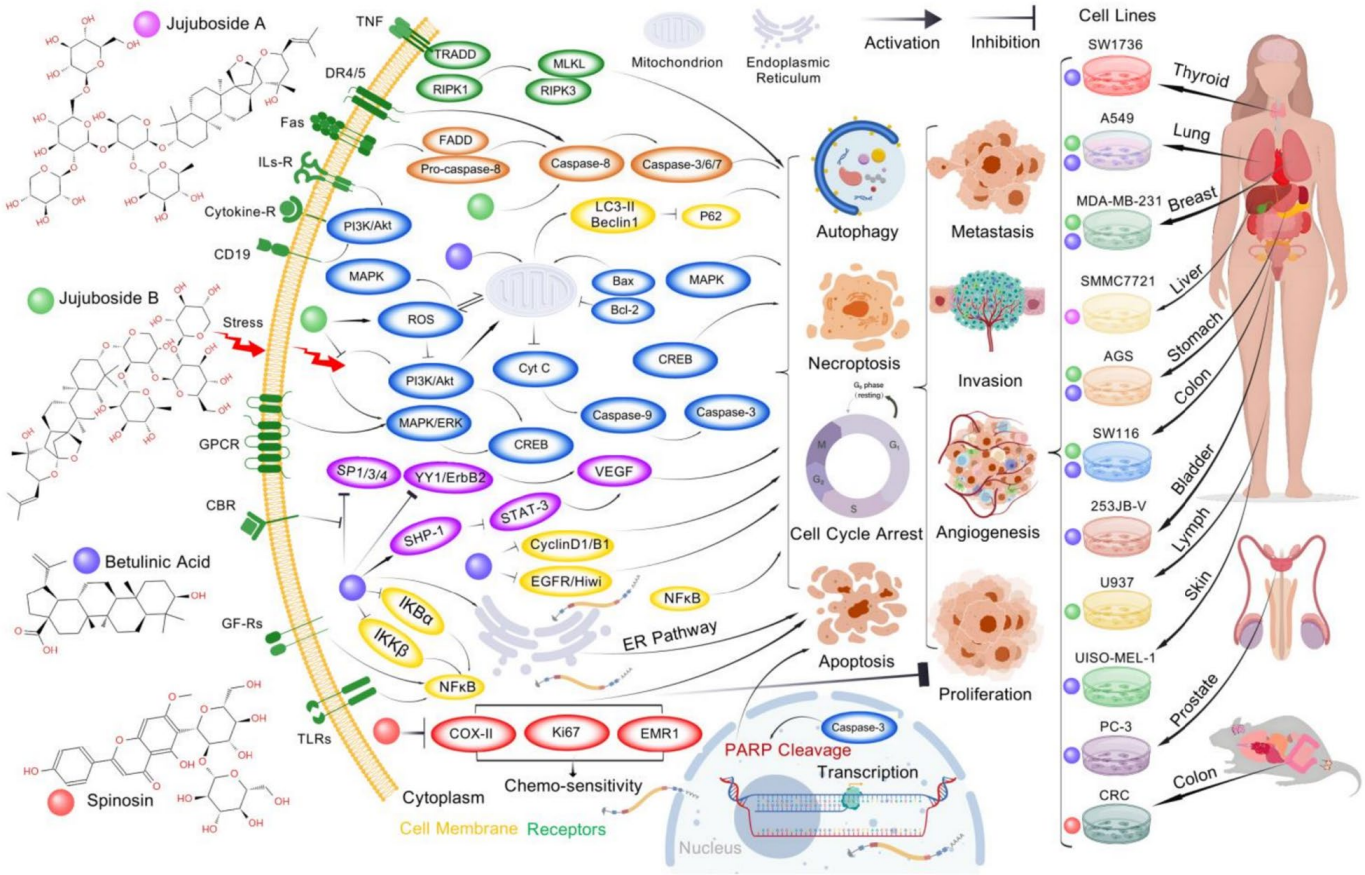
Molecular mechanisms underlying the antitumor pharmacodynamic effects of SJS active compounds. Created with BioGDP.com [22]

Emerging evidence highlights the multitargeted antitumor potential of jujuboside phytochemicals, particularly JuA/B, across various malignancies. The antitumor mechanisms of jujubosides suggest that JuA/B may serve as effective anticancer agents through integration of both in vitro and in vivo models. JuA demonstrates significant cytotoxic activity against human hepatocarcinoma SMMC-7721 cells, with a half-maximal inhibitory concentration (IC_50_) of 1.996 µg mL^−1^ [66]. In vitro analyses show that JuB triggers apoptotic and autophagic cell death pathways in AGS and HCT116 carcinoma cells, with corresponding in vivo efficacy observed by significant attenuation of HCT116 xenograft tumor progression in immunodeficient mice. JuB-induced apoptosis involves extrinsic pathways, augmented by Fas ligand (FasL) and caspase-8 activation [67]. After 24, 48, or 72 h of treatment, JuB (40, 80, or 120 µM) inhibited the growth of human histiocytic lymphoma U937 cells and exerted antileukemic effects by dose-dependently activating the receptor-interacting protein kinase 1 (RIPK1)/RIPK3/mixed lineage kinase domain-like pseudokinase (MLKL) pathway, inducing necroptosis. However, 120 µM JuB was potentially toxic to lymphoblastoid AHH-1 cells, with statistically significant differences at 160 µM [68]. In colorectal cancer cells (SW1116 and SW1463), JuB (40, 80, 120, 160, or 200 µM) administered for 12 h resulted in time- and dose-dependent antiproliferative activity, with no significant cytotoxicity in normal colon epithelial cells at concentrations ≤ 200 µM. JuB-triggered mitochondrial permeabilization in colorectal carcinoma cells involves redox signaling, as evidenced by the attenuation of the phosphoinositide 3-kinase/protein kinase B (PI3K/Akt) pathway and the accumulation of endogenous reactive oxygen species (ROS). A rigorously conducted 10-week preclinical investigation in murine colorectal cancer models showed that daily oral administration of JuB (40 mg kg^−1^ body weight) elicited dose-dependent suppression of tumor progression [69]. JuB also exhibits anticancer efficacy against breast carcinoma through induction of pro-autophagic and pro-apoptotic signaling cascades. Preclinical evaluation revealed that 48 h of exposure to JuB (25, 50, or 75 µM) resulted in concentration-dependent suppression of oncogenic proliferation in human breast carcinoma models (MDA-MB-231 and MCF-7 cells), along with dose-dependent inhibition of metastatic potential in MDA-MB-231 cells. In vivo evaluation in subcutaneous xenograft tumor models using MCF-7 and MDA-MB-231 cell lines, followed by single-dose oral administration of JuB (20, 30, or 40 mg kg^−1^) in tumor-bearing mice, revealed potent antineoplastic efficacy, as evidenced by tumor growth inhibition and the maintenance of physiological parameters, including body weight [70]. JuB treatment at 60 µM (IC_50_ ≈ 60 µM) significantly suppressed viability and colony formation in human lung cancer A549 cells by dephosphorylating the downstream signaling regulator CREB through blockade of the PI3K/Akt and MAPK/extracellular signal-regulated kinase (ERK) signaling pathways. In tumor-bearing mice subjected to chronic stress, daily intraperitoneal administration of JuB (40 mg kg^−1^) for two weeks inhibited tumor progression, demonstrating antitumor efficacy in both normatively fed tumor-bearing mice and chronic leukemia models. Mechanistically, JuB attenuated the phosphorylation-dependent activation of Akt/ERK/CREB/MAPK/PI3K cascade components, reduced pro-inflammatory cytokine levels, and markedly alleviated depression-like phenotypes in both tumor-bearing and non-tumor cohorts [71]. The molecular mechanisms underlying the antitumor pharmacodynamic effects of SJS-active compounds are depicted in Fig. 5.

#### Antitumor properties of SJS polyphenols, oils, and betulinic acid in colorectal carcinoma

Studies have shown that polyphenols, oils, and betulinic acid derived from SJS exhibit potent antitumor properties. Acid-hydrolyzed water-soluble polyphenolic fractions from SJS demonstrate significant antitumor effects against colorectal carcinoma (CRC), including inhibition of oncogenic hyperplasia, potentiation of intrinsic apoptosis, and increased chemosensitivity in CRC cells [72]. Betulinic acid, a bioactive constituent of SJS, also induces tumor cell death, further supporting the plant’s antitumor potential [73].

#### Summary: broad-spectrum anticancer mechanisms of SJS-derived compounds

Based on the integrated evidence, JuA/B and other SJS-derived compounds exert broad-spectrum anticancer activity through multimodal mechanisms, including the induction of apoptosis (extrinsic pathway via FasL/caspase-8), autophagy, and necroptosis (via RIPK1/RIPK3/MLKL). Their efficacy is consistently dose- and time-dependent across in vitro models of hepatocarcinoma, lymphoma, and colorectal, breast, and lung cancers and has been validated in multiple preclinical in vivo xenograft models through oral or intraperitoneal administration. Key mechanistic insights include the disruption of critical oncogenic signaling cascades (particularly the PI3K/Akt and MAPK/ERK pathways), induction of mitochondrial dysfunction and ROS accumulation, and modulation of inflammatory responses. Notably, JuB exhibits selectivity by exerting potent cytotoxicity in malignant cells while having minimal effects on normal epithelial cells at therapeutic concentrations. The additional antitumor properties of SJS polyphenols and betulinic acid further emphasize the plant’s potential as a source of multifaceted anticancer agents. These findings position jujubosides, particularly JuB, as promising candidates for further development, warranting investigation into their pharmacokinetics, safety profiles, and potential synergies with conventional therapies.

### Antioxidant activity

#### Antioxidant defense: fundamental mechanisms and the role of SJS

Antioxidant activity refers to the ability of compounds to neutralize ROS and free radicals, which cause oxidative damage to proteins, lipids, and DNA, contributing to diseases such as cancer, cardiovascular disorders, neurodegenerative diseases, and metabolic syndromes [74]. The body relies on both enzymatic and non-enzymatic antioxidants to maintain redox homeostasis and counteract oxidative stress. However, excessive ROS or impaired antioxidant systems can overwhelm cellular defenses, leading to disease progression. SJS functions as a multiconstituent antioxidant defense system, with its diverse phytochemicals collectively mitigating oxidative stress through complementary radical-scavenging and enzymatic mechanisms. Redox reactions are fundamental to nearly all biological processes, and harmful free radicals generated during metabolism are implicated in aging and the pathogenesis of various diseases.

#### Broad-spectrum antioxidant properties of SJS constituents

Recent research has focused on natural antioxidants, with SJS demonstrating broad antioxidative properties attributed to its saponins, flavonoids, oils, phenolic acids, polysaccharides, and alkaloids [75]. Capillary electrophoresis analysis revealed synergistic superoxide anion radical (O₂^−^·)-scavenging effects between SOD and JuA/B in serum matrices. JuB exhibited an IC_50_ of 31.6 mg kg^−1^ in 60 µL of serum medium, with dose-responsive radical-scavenging activity observed at concentrations of 20–40 mg·L^−1^. While SOD synergizes with JuA/B to exhibit antioxidant effects, no clear dose-dependent relationship was established [76]. Comparative quantification of bioactive components (JuA/B and betulinic acid) in SJS extracts, using vitamin C as a positive control, demonstrated potent in vitro antioxidative capacity, including total reducing power, hydroxyl radical (·OH) scavenging, and 1,1-diphenyl-2-picrylhydrazyl (DPPH) radical inhibition, confirming that SJS is a robust antioxidant source [7]. Betulinic acid from SJS significantly suppressed SOD and endothelial nitric oxide synthase (eNOS) enzymatic activities in aortic tissues from spontaneously hypertensive rats, further validating its antioxidative efficacy [77].

#### Hepatoprotective and enzymatic antioxidant enhancement by SJS flavonoids

Flavonoid extracts obtained via ultrasound-assisted extraction (UAE) exhibited superior scavenging capacities against ABTS^+^, DPPH·, O₂^−^·, and ·OH radicals, alongside reduced ROS accumulation in PC12 cells [78]. A 30-day oral gavage protocol employing flavonoids (200–800 mg kg^−1^) and vitamin C (100 mg kg^−1^) in murine models of hepatic injury resulted in marked attenuation of hepatic MDA, alanine aminotransferase (ALT), and aspartate aminotransferase (AST), while concurrently augmenting total antioxidant capacity (T-AOC), GPx, CAT, and SOD, indicating hepatoprotective effects against oxidative damage [79].

#### Optimization of antioxidant efficacy: extraction methods

UAE-derived SJS oil outperformed oils extracted by heat reflux in terms of ABTS^+^, O₂^−^·, and ·OH radical scavenging [80]. Compared with hot water extraction (HWE), polysaccharides extracted via UAE and HWE showed in vitro antioxidative effects against ABTS^+^, O₂^−^·, ·OH, and ferrous ions, with UAE polysaccharides demonstrating stronger ·OH scavenging, Fe^2+^ chelation, and cyclooxygenase-2 (COX-2) induction in RAW264.7 cells [81]. Magnoflorine, a principal alkaloid in SJS, upregulated the expression of antioxidant stress-related proteins such as phospholipid complexes [65].

#### Summary: SJS as a multifaceted antioxidant system with integrated defense mechanisms

In conclusion, SJS functions as a multicomponent antioxidant system. Its efficacy arises from a broad spectrum of bioactive compounds—saponins (JuA/B), flavonoids, oils, phenolic acids, polysaccharides, and alkaloids (e.g., magnoflorine)—each contributing distinct radical-scavenging capacities against O₂^−^·, ·OH, DPPH, ABTS^+^, and ferrous ions. Notably, extraction methodology significantly influences antioxidant potency, with UAE consistently yielding flavonoid and polysaccharide fractions with superior activity compared to traditional methods. Beyond direct radical neutralization, SJS components enhance endogenous antioxidant defenses, as evidenced by increased T-AOC, GPx, CAT, and SOD activities and reduced ROS/MDA levels in cellular and hepatic injury models. Synergistic interactions, such as those between JuA/B and endogenous SOD, further amplify protection. Critically, these antioxidant actions translate into functional benefits: betulinic acid modulates key enzymes (SOD, eNOS) in the hypertensive vasculature, flavonoids mitigate hepatic oxidative damage and enzyme leakage, and polysaccharides influence inflammatory pathways (COX-2 induction). The collective data highlight that SJS orchestrates a multilayered defense against oxidative stress through direct scavenging, enzymatic upregulation, metal chelation, and potential anti-inflammatory crosstalk. This positions SJS as a potent natural source for mitigating oxidative damage across multiple organ systems.

### Cardioprotective effects

#### Key mechanisms and therapeutic strategies

Cardioprotective effects refer to therapeutic strategies aimed at preventing or mitigating myocardial injury caused by ischemia, reperfusion, or excessive neurohormonal activation. Effective cardioprotection involves multi-tiered interventions that counter ischemic damage, stabilize electrophysiological activity, and enhance contractile performance [82]. Key mechanisms include reducing infarct size, modulating apoptotic pathways (e.g., regulation of B-cell lymphoma 2 (Bcl-2) and Bcl-2-associated X (Bax) proteins), and activating pro-survival signaling cascades, such as PI3K/Akt/mTOR and MAPK/Akt. Additionally, calcium current modulation contributes to antiarrhythmic outcomes, while positive inotropic effects mediated by calcium-dependent pathways support functional recovery.

#### Multifaceted cardioprotective effects of SJS bioactives: from ischemia to arrhythmia

Direct cardioprotective effects of SJS extract and its bioactive components have been consistently observed in various preclinical models. SJS and its bioactive constituents demonstrate cardioprotective efficacy through multi-tiered interventions that address ischemic injury, arrhythmogenesis, and contractile dysfunction. Total jujuboside extract has shown potent cardioprotective effects. Prophylactic administration of *Ziziphus jujuba* saponins significantly reduced myocardial infarction size in a LAD ligation model of rats, protected against ischemic injury, decreased heart rate, and improved electrocardiogram parameters (S-T segment and T-wave elevation) during acute myocardial ischemia [83]. These saponins also counteract pituitary-induced myocardial ischemia in rats [84]. In H9C2 cardiomyocytes, exposure to isoproterenol (ISO) induced cellular injury, which was followed by JuA pretreatment to assess its protective effects on cell viability, morphological alterations, and activation of the PI3K/Akt/mTOR signaling axis. These results highlight the cardioprotective potential of JuA against ISO-induced cardiomyocyte injury through activation of PI3K/Akt/mTOR survival pathways [85]. Furthermore, JuA demonstrated anti-apoptotic effects in H9c2 cardiomyoblasts exposed to norepinephrine via modulation of MAPK/Akt signaling cross-talk [86]. Whole-cell patch-clamp analysis of rat ventricular cardiomyocytes revealed JuA-mediated regulation of the L-type calcium current in a concentration-dependent manner [87]. Further mechanistic investigations demonstrated that JuA protects against ischemia/reperfusion (I/R)-induced ventricular arrhythmias in rodent models, along with amelioration of I/R-associated downregulation of anti-apoptotic Bcl-2 protein and inhibition of pro-apoptotic Bax protein upregulation in cardiac myocytes [88].

#### SJS fatty acids exert calcium-dependent positive inotropic effects *in *ex vivo hearts

SJS-derived fatty acids exhibited significant cardiotonic activity *in *ex vivo toad heart preparations. Dose–response analysis, spanning a concentration range from 5 × 10⁻^3^ mL·10 mL⁻^1^ (baseline) to 2 × 10⁻^2^ mL·10 mL⁻^1^ (maximum tested dose), showed that these compounds induce positive inotropic effects that correlate with extracellular calcium levels, likely mediated through accelerated Ca^2^⁺ influx [89].

#### Summary: integrated cardioprotection by SJS saponins and fatty acids through complementary mechanisms

In conclusion, SJS constituents, particularly saponins and fatty acids, provide multifaceted cardioprotection through distinct yet potentially complementary mechanisms. Saponins in SJS mitigate ischemic damage at both the organ level (reduced infarction area and improved electrocardiogram parameters) and the cellular level (activation of anti-apoptotic PI3K/Akt/mTOR and MAPK/Akt survival pathways), while also stabilizing electrophysiological activity through calcium current modulation and antiarrhythmic Bcl-2/Bax rebalancing. Similarly, SJS fatty acids enhance contractile function via calcium-dependent inotropic effects, demonstrating a comprehensive therapeutic profile that spans structural integrity preservation, survival pathway activation, and functional performance optimization.

### Antidyslipidemic and antihypertensive effects

#### Cardioprotective effects of SJS: multifactorial intervention in atherogenic pathways and eNOS recoupling

In addition to its previously described activities, SJS extract and its constituents demonstrate significant cardioprotective effects through multifactorial interventions in key atherogenic and vascular dysfunction pathways. Preventing foam cell formation is a critical therapeutic target in atherosclerosis. SJS extract markedly suppressed acetylated low-density lipoprotein (LDL)-induced foam cell formation, suggesting that its triterpenoids may offer anti-atherogenic benefits [90]. Cardiovascular disorders are often exacerbated by endothelial nitric oxide synthase (eNOS) dysfunction, which involves enzymatic uncoupling and reduced nitric oxide (NO) bioavailability due to oxidative stress-induced post-translational modifications. Therapeutic strategies targeting eNOS recoupling and transcriptional upregulation are promising for vascular protection.

#### Dual regulation of endothelial function by SJS

In vitro studies using human umbilical vein endothelial cells and EA.hy 926 endothelial cell lines showed that SJS extract exerts a dual regulatory effect on eNOS expression. Dose- and time-dependent increases in eNOS promoter activity, mRNA transcription, protein levels, and functional NO output were observed. Betulinic acid from SJS demonstrated dual functionality—upregulating eNOS and downregulating nicotinamide adenine dinucleotide phosphate (NADPH) oxidase activity—further highlighting its cardiovascular therapeutic potential [91]. In a model of Nω-nitro-L-arginine methyl ester (L-NAME)-induced hypertension, betulinic acid exhibited antihypertensive effects, accompanied by the restoration of acetylcholine-mediated endothelium-dependent vasorelaxation. This suggests a therapeutic mechanism involving redox modulation and preservation of NO signaling integrity in the vascular system [77].

#### Pleiotropic vascular-metabolic regulation by SJS

SJS constituents, particularly betulinic acid, act as multinodal orchestrators by simultaneously modulating atherogenic lipid processing (inhibiting foam cell formation), maintaining redox homeostasis in the vascular endothelium (via NADPH oxidase suppression and eNOS upregulation, thereby increasing NO availability), regulating ion channel dynamics, and facilitating inflammation resolution. This convergence of lipid metabolism, oxidative stress, and calcium signaling networks positions SJS as a pleiotropic modulator at the vascular–metabolic interface, with translational potential for mitigating atherosclerosis, vascular remodeling in hypertension, and microvascular complications in diabetes.

### Immunomodulatory effects

#### Immunomodulation fundamentals and SJS macromolecules as therapeutic agents

Immunomodulatory effects involve regulating immune activity to either enhance or suppress immune responses. Dysregulation of these processes can lead to autoimmune diseases or chronic inflammation [92]. Key pathways, such as NF-κB and MAPK, control cytokine production and immune cell activation. An imbalance in these pathways can result in excessive inflammation. Additionally, the gut, through gut-associated lymphoid tissue and the microbiota, plays a crucial role in immunity. As a result, therapies targeting gut immunity, barrier function, and microbial communities hold significant therapeutic potential for inflammatory and autoimmune conditions [93]. Emerging evidence further highlights the immunoregulatory mechanisms of SJS-derived macromolecules, demonstrating their capacity to modulate both systemic immune responses and gut mucosal immunity through interconnected signaling and microbiota-mediated pathways.

#### SJS protein (SJSP): immunostimulation via NF-κB and MAPK pathway regulation

SJSP exhibits immunostimulatory effects in cyclophosphamide (CTX)-induced immunosuppressed mice by enhancing immune organ development, peritoneal macrophage phagocytosis, and T lymphocyte subset differentiation, thereby ameliorating systemic immunosuppression. SJSP exerts immunomodulatory effects through dual regulation of the NF-κB and MAPK pathways. This is evident from changes in the phosphorylation dynamics of phospho-inhibitor of κB alpha (p-IκBα), phospho-c-Jun N-terminal kinase (p-JNK), and phospho-extracellular signal-regulated kinase (p-ERK). These findings suggest context-dependent signaling bias: while NF-κB suppression may mitigate pro-inflammatory cytokine storms, MAPK activation could promote T-cell receptor signaling and T helper cell (Th) 1/Th17 polarization. The purified S1F2G1 fraction demonstrated marked immunomodulatory activity, further validating SJSP’s potential in managing immunosuppression [94].

#### SJS polysaccharides: attenuating colitis via gut microbiota reshaping and barrier enhancement

In a study using C57BL/6 mice, daily oral gavage of SJS polysaccharides at a dose of 100 mg·kg^−1^ body weight for 4 weeks significantly reduced spinosin absorption compared to controls. Additionally, the expression of occludin, multidrug resistance-associated protein 2 (MRP2), and P-glycoprotein (P-gp) was markedly increased in the colon. These polysaccharides also reshaped the gut microbiota composition, decreasing the abundance of *Bacteroidetes* and increasing the abundance of *Firmicutes* and *Verrucomicrobia*. Furthermore, they regulated tight junction protein and efflux transporter expression in Caco-2 cells, although the intestinal microbiota culture supernatants showed no comparable bioactivity. Colonic histopathology revealed significant attenuation of trinitrobenzene sulfonic acid (TNBS)-induced experimental colitis, as indicated by diminished leukocyte infiltration in the lamina propria and submucosal layers. NF-κB/MAPK signaling dynamics orchestrated this immunomodulatory response in murine RAW264.7 macrophages [9].

Collectively, these findings position SJS macromolecules as multimodal immunoregulators capable of addressing immune dysfunction through direct signaling pathway modulation in immune cells, reinforcement of intestinal barrier integrity, and strategic reshaping of the gut microbiota.

### Other effects

SJS and its isolated compounds, particularly JuA, exhibit broad-spectrum pharmacodynamic activities across various experimental models, as demonstrated by several key findings. SJS oil suppressed 12-O-tetradecanoylphorbol-13-acetate-induced cutaneous inflammation in experimental mice [95]. Dammarane-type saponins from SJS moderately inhibited lipopolysaccharide-induced TNF-α release in RAW 264.7 macrophages [96]. JuA promoted proliferation, suppressed apoptosis, and enhanced osteogenic differentiation in murine MC3T3-E1 preosteoblasts during in vitro osteogenesis [97]. In diabetic rats, JuA attenuated glomerular apoptosis by modulating the intrinsic mitochondrial pathway and transforming growth factor-beta 1 (TGF-β1) transcriptional activity [98].

JuA has shown potent antiplatelet activity in rabbit models, inhibiting platelet aggregation induced by arachidonic acid, adenosine diphosphate (ADP), and platelet factor 4 (PF-4), while reducing serum levels of platelet-activating factor (PAF), P-selectin, and PF-4 [99]. Its melanogenesis-inhibitory effects are attributed to tyrosinase suppression [100]. SJS extract reduced body temperature and enhanced weight gain in heat-stressed poultry while preventing NMDA-induced epileptogenesis through calcium influx blockade [101]. Further studies revealed that JuA prevents kainic acid-induced seizures by increasing intracellular chloride concentration and lowering calcium levels [102].

This collective evidence positions SJS and JuA as multitarget therapeutic candidates with significant potential for managing inflammation, tissue degeneration, thrombotic events, metabolic stress, and neurological hyperexcitability.

## Pharmacokinetics

### Flavonoids

#### Pharmacokinetic profiling of SJS flavonoids in beagle dogs

Flavonoids are key bioactive components in SJS, and pharmacokinetic studies on the absorption, distribution, metabolism, and excretion of these compounds highlight the role of structural factors, microbial interactions, and formulation-dependent bioavailability. A validated ultrahigh-performance liquid chromatography-tandem mass spectrometry (UPLC-MS/MS) analytical platform was developed to simultaneously analyze nine components in beagle dog plasma following the administration of fried SJS extract. After a single dose, the time to reach the maximum plasma concentration (*T*_*max*_) of 6‴-feruloylspinosin was significantly earlier compared to isospinosin and spinosin, indicating a faster absorption rate of 6‴-feruloylspinosin. The maximum plasma concentrations (*C*_*max*_) of isospinosin, spinosin, and 6‴-feruloylspinosin were 13.0, 31.5, and 4.62 ng mL⁻^1^, respectively, with elimination half-lives (*t*_*1/2*_) of 4.23, 4.31, and 2.08 h, respectively. In comparison, isospinosin and spinosin had greater in vivo exposure and slower elimination, whereas the *t*_*1/2*_ of vicenin II in beagle dogs was 6.56 h, indicating a longer duration of pharmacodynamic effects [103].

#### Elimination pathways and gut microbiota-mediated biotransformation of SJS flavonoids in rats

Pharmacokinetic profiling of total flavonoids from SJS in Sprague–Dawley (SD) rats revealed that the flavonoid-enriched fraction was predominantly eliminated via renal and fecal routes as intact parent compounds, including characteristic flavonoid glycosides and 6‴-feruloyl-substituted jujuboside derivatives, with minor metabolic conversion to secondary glycosylated conjugates [18]. In vitro studies indicated that β-glucosidase from the rat gut microbiota catalyzes the deglycosylation of spinosin derivatives, including 6‴-feruloylspinosin and 6‴-*p*-coumaroylspinosin, yielding spinosin as the final product [51]. The intestinal microbiota also selectively cleaves aromatic acyl groups (e.g., *p*-coumaroyl, feruloyl, and *p*-hydroxybenzoyl) from spinosin derivatives, resulting in the formation of spinosin through microbial biotransformation. Subsequent enzymatic hydrolysis of spinosin by gut-derived glycosidases then facilitates the release of swertisin, a 6-C-linked flavonoid glycoside. In a rat gastrointestinal absorption model, spinosin was absorbed throughout the gastrointestinal tract via a first-order kinetic process, with passive diffusion as the absorption mechanism. Absorption efficacy followed the order: duodenum > colon > jejunum > ileum, with the stomach showing the best absorption [104].

#### Modulation of spinosin disposition by drug interactions and comparative tissue distribution in rat models

Pharmacokinetic studies of spinosin in the brain, plasma, and bile, using an integrated UPLC-MS/MS and in vivo microdialysis sampling platform, showed that cyclosporine A significantly modulated spinosin’s systemic and CNS disposition, altering absorption kinetics, distribution patterns, and biliary excretion rates under co-administration conditions [105]. Peak spinosin concentrations in the rat brain and liver at 20 min post-oral gavage were as follows: the liver had the highest tissue accumulation (*C*_*max*_ = 220.3 ± 4.37 µg g⁻^1^), followed by the kidneys (90.82 ± 3.29 µg g⁻^1^), plasma (37.64 ± 4.85 µg mL⁻^1^), and spleen (29.86 ± 5.22 µg g⁻^1^), while the testicular and brain concentrations were minimal. No spinosin distribution was observed in smooth muscle or skeletal muscle [106]. In a study measuring spinosin in the plasma of normal and insomnia rats, the absorption rate in insomnia rats was significantly lower compared to normal rats [107].

#### Brain penetration and formulation-dependent bioavailability of spinosin

Following intravenous administration in rats, spinosin was widely distributed and rapidly translocated throughout the body, with extensive penetration into key brain structures, including the limbic (hippocampus, olfactory bulb), neostriatal regions, and cerebellar circuits. Spatial pharmacokinetic analysis showed selective accumulation in the dorsal striatum and CA1 hippocampal subfield [108]. Spinosin bioavailability was significantly lower in monotherapy regimens compared to SJSD formulations, with plasma levels remaining below detection limits under identical dosing conditions. This formulation-dependent difference suggests that herb–herb interactions within SJSD synergistically enhance spinosin absorption, profoundly modulating its systemic exposure profile [109].

#### Comparative pharmacokinetics of spinosin: delayed absorption in insomnia model

After intragastric administration of SJSD, spinosin exhibited a mean *T*_*max*_ of 6.00 ± 0.63 h and a *t*_*1/2*_ of 5.02 ± 0.43 h. A comparative pharmacokinetic analysis in healthy and insomnia-induced rats showed reduced spinosin and mangiferin absorption in the insomnia group. Specifically, normal rats had a spinosin *T*_*max*_ of 0.74 ± 0.11 h and a *t*_*1/2*_ of 4.4 ± 1.0 h, while insomnia rats exhibited a prolonged *T*_*max*_ of 1.4 ± 0.6 h and a similar *t*_*1/2*_ of 4.3 ± 1.3 h [107].

**Fig. 6:**
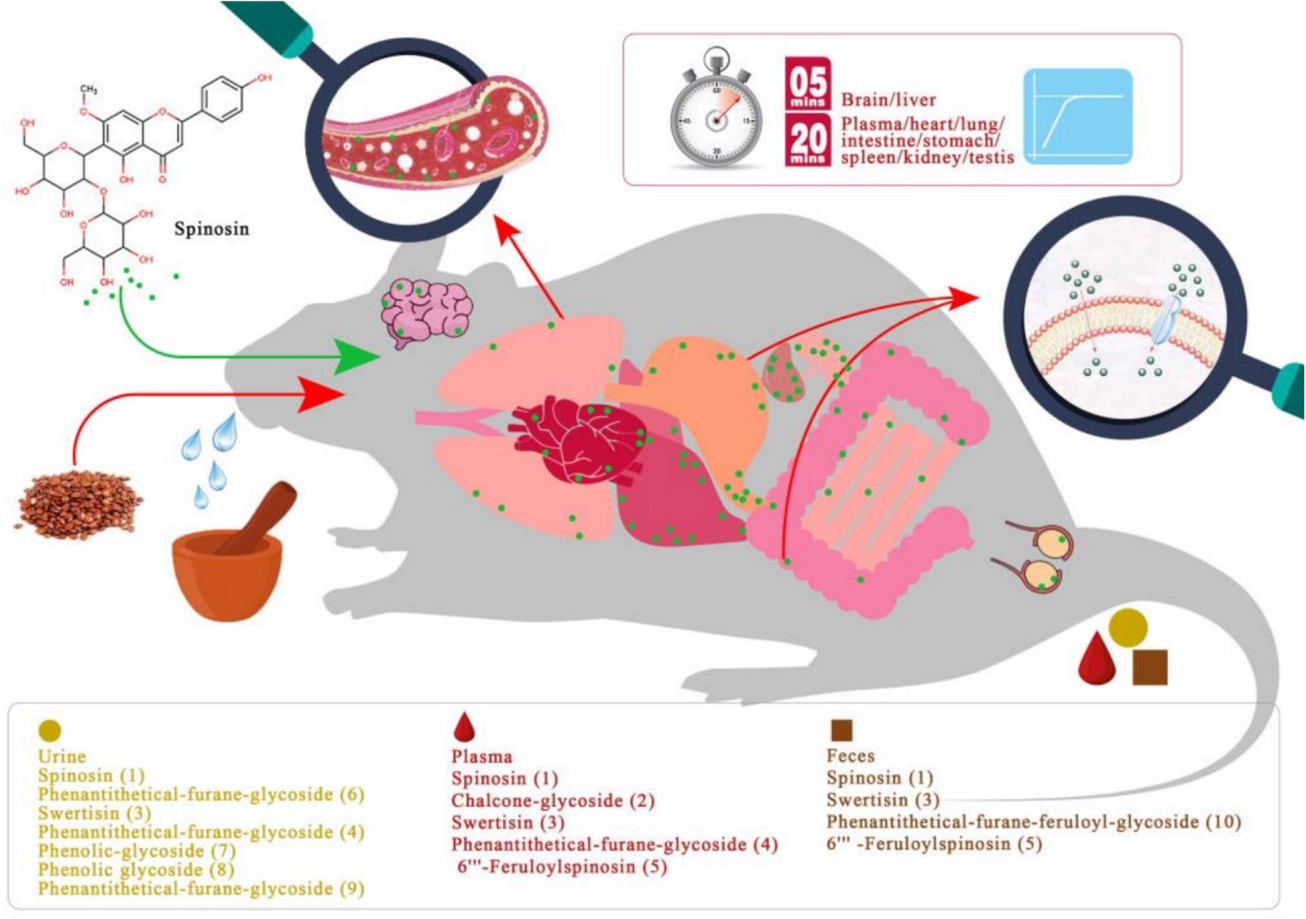
Pharmacokinetic schematic diagram of spinosin

These findings emphasize the critical interplay between chemical structure, gut microbiota-mediated biotransformation, and pathological conditions in shaping the pharmacokinetics of SJS-derived flavonoids, with implications for formulation design and personalized therapeutic strategies. A pharmacokinetic schematic diagram of spinosin is presented in Fig. 6.

#### A complex pharmacokinetic-efficacy network for SJS flavonoids

Based on comprehensive pharmacokinetic data from multiple studies, a complex and interconnected regulatory network likely governs the efficacy of SJS flavonoids. This network is influenced by structural modifications, gut microbiota interactions, formulation effects, and pathophysiological states. The characteristic acylated flavonoid glycosides (e.g., 6‴-feruloylspinosin and 6‴-*p*-coumaroylspinosin) act as natural prodrugs, which undergo rapid initial absorption and extensive gut microbial biotransformation, both of which are essential for their efficacy. Gut microbiota enzymes selectively cleave aromatic acyl groups and glycosidic bonds, converting these complex precursors first into spinosin and ultimately into the core aglycone, swertisin. This stepwise microbial metabolism shapes the systemic exposure profile of bioactive metabolites, potentially targeting specific tissues or enabling prolonged effects.

#### Gut microbiota-mediated biotransformation as a key metabolic pathway

Spinosin is absorbed throughout the gastrointestinal tract via passive diffusion, leading to widespread tissue distribution. However, it faces significant barriers to penetrating the CNS under normal physiological conditions [104]. Its efficacy, particularly for CNS-related conditions like insomnia, is highly influenced by formulation. The SJSD multi-herb formulation significantly enhances spinosin bioavailability and systemic exposure compared to monotherapy, likely through herb–herb interactions that inhibit efflux transporters or increase solubility and permeability.

#### Formulation-dependent absorption and tissue distribution of spinosin

Pathological conditions such as insomnia disrupt this network, reducing spinosin’s absorption rate and delaying *T*_*max*_ [107]. This suggests that disease-altered gut function or transporter expression may affect bioavailability. Additionally, the extensive renal and fecal excretion of intact parent glycosides and acylated derivatives indicates a potential enterohepatic recycling loop or colonic targeting for local effects. The selective tissue accumulation and specific brain region penetration under facilitating conditions suggest that transporter-mediated distribution mechanisms regulate flavonoid concentrations at therapeutic sites.

#### Integrated network model of SJS flavonoid efficacy

Thus, the pharmacodynamic effect of SJS flavonoids emerges from a dynamic interplay: the structural features of precursor molecules dictate their metabolic fate via the gut microbiota; the resulting metabolites and their systemic exposure are amplified or restricted by formulation synergies and pathological conditions; and tissue-specific transporter systems regulate access to therapeutic targets. This sophisticated pharmacokinetic–efficacy network highlights the key regulatory roles of microbial biotransformation, herb–herb interactions, and disease-specific modulation.

### Triterpenoid saponins

#### Gut microbiota-driven biotransformation and metabolic pathways of jujubosides

Recent pharmacokinetic studies of jujuboside A/B (JuA/B) from SJS have clarified their metabolic trajectories and systemic behaviors, highlighting the key roles of gut microbiota-driven biotransformation and structural modifications in determining oral bioavailability. The results indicate that JuA components in SJS can exist as prototypes or form glycosaminoglycans or saponins through sugar chain removal [110]. Pharmacokinetic evaluation of JuA in murine plasma, using astragaloside IV as the internal standard, revealed rapid systemic absorption followed by prolonged metabolic elimination [111]. When gastric contents from Wistar rats were incubated at 37 °C with a pharmacodynamically relevant concentration of JuA, the compound was significantly degraded. Three metabolites, primarily hydrolysis products of JuA, were identified in the samples [112]. In addition to the presence of prototype substances, metabolites such as spironolactone and 6‴-ferulic acid spironolactone were detected in plasma, urine, and feces following oral dosing of SJS extract in rats. Notably, 6‴-ferulic acid spironolactone removes ferulic acid and converts it into spironolactone, which further removes one glucose molecule and converts it into amphotericin. Other decarboxylated products at the C4 position and their further metabolites were also observed [113].

#### Gut microbiota hydrolysis and limited bioavailability of jujubosides

**Fig. 7:**
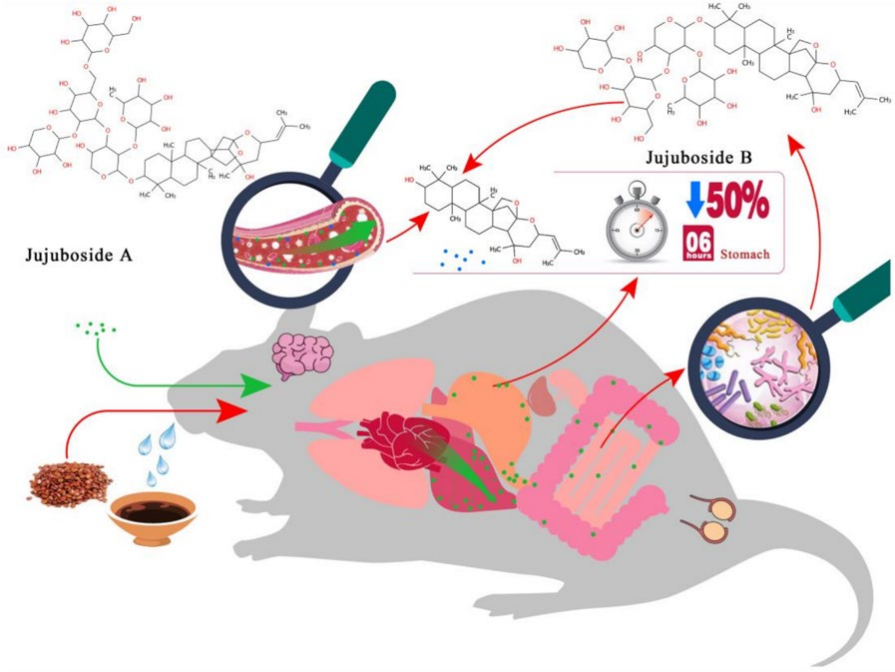
Pharmacokinetic schematic diagram of jujuboside (**A**) and jujuboside (**B**)

HPLC–MS/MS analysis of JuA in rat plasma showed a 40–4000 ng mL⁻^1^ linear dynamic range. Absolute bioavailability was found to be only 1.32% [114]. In vitro incubation of JuB with gut microbiota, analyzed via rapid resolution liquid chromatography (RRLC)-MS/MS, demonstrated first-order kinetic degradation, producing hydrolysis products as primary metabolites [115]. JuB exhibited one-compartment kinetics following oral administration and two-compartment kinetics following intravenous administration in rats, with a low systemic exposure of 3.6% absolute oral bioavailability [103]. The pharmacokinetic processes of JuB fit a one-compartment model for oral administration and a two-compartment model for intravenous administration, with a minimal *t*_*1/2*_ consistent with literature reports [116]. It is hypothesized that JuB undergoes hydrolysis by the intestinal microbiota, which further metabolizes it into aglycones [117]. A pharmacokinetic schematic diagram of JuA/B is presented in Fig. 7.

#### Metabolic-efficacy network of jujubosides A/B governed by hydrolysis and clearance

Based on emerging pharmacokinetic data for JuA/B in SJS, a sophisticated metabolic-efficacy network can be proposed. Structural lability, microbial biotransformation, and metabolic clearance together govern their bioavailability and therapeutic potential. JuA/B function as structurally labile prodrugs, with their therapeutic effects tightly regulated by sequential hydrolysis. Gastric acid and enzymes initiate degradation into primary hydrolysis products, but gut microbiota-derived hydrolytic enzymes drive critical biotransformation. This microbial processing acts as a metabolic gateway, progressively stripping sugar chains and converting the compounds to their core aglycones. This extensive hydrolysis generates bioactive metabolites but significantly limits bioavailability, as demonstrated by the low absolute oral bioavailability primarily due to presystemic degradation by the intestinal microbiota and gastric processes [103, 114]. The resulting metabolites undergo rapid phase I metabolism, leading to accelerated systemic clearance and diverse metabolite profiles in plasma, urine, and feces [113].

#### The dual role of gut microbiota: bioactivation versus bioavailability barrier for jujubosides

Crucially, this network suggests that the ultimate pharmacodynamic effects likely stem from the cascade of hydrolysis and phase I metabolites, rather than from the parent JuA/B compounds themselves. These metabolites, with altered physicochemical properties due to increased lipophilicity or reduced molecular weight, may exhibit increased membrane permeability or greater affinity for specific cellular targets. However, their rapid clearance requires innovative delivery strategies, such as microbiota-protective formulations, targeted colonic release, or co-administration with metabolic inhibitors, to optimize their therapeutic potential. Therefore, efficacy arises from a delicate balance: microbial and host enzymatic hydrolysis activates latent pharmacophores within the complex saponin structures, but simultaneous presystemic and systemic metabolic clearance restricts systemic exposure. This positions the gut microbiota as both a key bioactivator and a primary barrier to oral bioavailability.

## Clinical applicability

### Applications

Insomnia and comorbid neuropsychiatric disorders represent a global health burden, impacting cognitive performance, cardiometabolic health, and quality of life, with conventional pharmacotherapies often limited by tolerability concerns and dependence risks. In TCM, SJS, a cornerstone herb in classical formulations such as SZRD, has been utilized for millennia to address sleep disturbances and associated symptoms. Recent decades have witnessed an expansion of clinical inquiry into its broader therapeutic potential, encompassing not only sleep improvement but also anxiety, depression, cognitive dysfunction, and metabolic comorbidities. This section synthesizes clinical evidence on SJS or SJS-based formulas for insomnia and other conditions.

#### Insomnia

SJS demonstrated significant short-term improvements in subjective sleep quality across multiple RCTs. Compared to placebo, it showed a standardized mean difference of – 0.58 (95% confidence interval (CI) [– 1.04, – 0.11], *P* < 0.01) in combined insomnia/sleep disturbance populations [118]. When compared to benzodiazepines or cognitive behavioral therapy, SJS reduced insomnia severity by 2.68 points (95% CI [–5.50, – 0.22], *P* = 0.03) at 4 weeks [118]. Meta-analysis of 12 RCTs (n = 1311) revealed SZRD alone or combined with Western medicine improved clinical effective rate (relative risk (RR) = 1.22, 95% CI [1.16, 1.29], *P* < 0.00001) and reduced recurrence rate (RR 0.47, 95% CI [0.28, 0.80], *P* = 0.005) [119]. Notably, SZRD showed superiority in slow-wave sleep maintenance (N3 stage duration) compared to estazolam (*F* = 4.98, *P* = 0.029) [120]. Safety profiles were favorable, with adverse event rates of 6–22% versus 22–30% in control groups [121, 122].

#### Anxiety and depression

SJS Tang significantly reduced Hamilton anxiety scale scores by 30% (*P* < 0.05) and improved daytime dysfunction in menopausal women (Pittsburgh Sleep Quality Index 13.0 ± 2.9 to 9.0 ± 3.2, 95% CI [– 4.93, – 3.10]) [123, 124]. Pharmacological studies identified neurotransmitter regulation as key mechanism, including GABAergic (jujuboside A, spinosin) and serotonergic (5-HT) pathway modulation [123, 125]. Network pharmacology confirmed neuroactive ligand-receptor interaction pathways, with 5-HT1A/2A and GABAA receptor expression changes [126].

#### Cognitive impairment and stroke recovery

Modified SZRD improved cognitive function (*P* = 0.006) and reduced cortisol levels (*P* = 0.036) in post-stroke patients, with superior effects on depression scores (*P* = 0.034) compared to zolpidem [127]. In chronic insomnia disorder, SZRD combined with lorazepam showed higher Insomnia Severity Index reduction rates at weeks 8 and 12 (*P* < 0.05), with fewer adverse effects [122]. Functional magnetic resonance imaging studies revealed SZRD-specific regional homogeneity changes in the middle occipital gyrus, correlating with cognitive improvement [11].

#### Perioperative and comorbid conditions

SJS Decoction combined with continuous iliac fascia space block reduced postoperative delirium incidence (4% vs 20%, *P* < 0.05) and adverse reactions (6% vs 22%, *P* < 0.05) in hip surgery patients [121]. In perimenopausal insomnia, Jiawei SJS Tang normalized estradiol/follicle-stimulating hormone/luteinizing hormone levels and upregulated hypothalamic 5-HT/GABAA receptor expression (*P* < 0.05) [126].

#### Methodological considerations

Despite positive findings, current evidence is limited by small sample sizes (median n = 785 across RCTs) and variable study quality (Cochrane scores ≤ 3/8 in early trials) [128]. Ongoing trials are evaluating SZRD versus estazolam in chronic insomnia disorder with 20-week follow-up [120]. Future research should prioritize large-scale RCTs with objective measures and mechanistic studies on Q-markers (jujuboside A, spinosin) [129, 130].

SJS exhibits multidimensional efficacy in neuropsychiatric disorders through GABAergic/serotonergic modulation, anti-inflammatory effects, and neuroendocrine regulation. While short-term benefits are well-documented, long-term safety and efficacy require validation through high-quality RCTs. The identified Q-markers provide a foundation for quality control in herbal preparations, supporting its clinical application as an evidence-based alternative to conventional therapies.

### Safety

SJS has a well-established reputation for safety as a traditional Chinese medicine. Classified among China’s first dual-use medicinal and edible substances by the Ministry of Health, its safety is supported by minimal toxic reactions reported in both dietary and clinical applications. According to classical pharmacopeia, SJS exhibits toxicity only at exceptionally high doses, with experimental studies across multiple models consistently confirming its low toxicity.

Notably, a compound capsule containing *Gastrodiae Rhizoma* and SJS, tested at a maximum dose of 30,000 mg kg^−1^ via gavage, showed no toxicity or mortality [131]. Wang et al. evaluated an alcohol extract of SJS via intravenous injection and reported an LD_50_ of 27.5 g kg^−1^ (95% CI [25.1, 30.1]). While intravenous administration induced toxic reactions and mortality in some animals, no histopathological changes were detected in major organs. Critically, intragastric administration at 340 g kg^−1^ caused no deaths or significant toxicity, emphasizing the extract’s minimal oral toxicity [132]. Chronic toxicity tests in rats further corroborated SJS’s exceptional safety. Similarly, acute and subchronic evaluations of SJS oral liquid (500 mg mL^−1^) in chickens revealed no adverse effects, with a maximum tolerance dose exceeding 20 g kg^−1^. Biochemical alterations observed during subchronic testing were reversible, supporting the safety of prolonged oral use [133]. Genetic toxicity assessments, including the Ames test, mouse bone marrow micronucleus test, and sperm deformity test, all returned negative results, indicating no genotoxic potential within the tested scope [131].

While data on the toxicity of SJS extracts and specific constituents are limited, accumulated evidence from acute, subchronic, and genetic toxicological studies consistently demonstrates very low toxicity. Its favorable safety profile, particularly via oral administration, supports its clinical applicability and alignment with traditional use.

## Discssion

### Multicomponent crosstalk and synergistic therapeutic effects in SJS

**Fig. 8:**
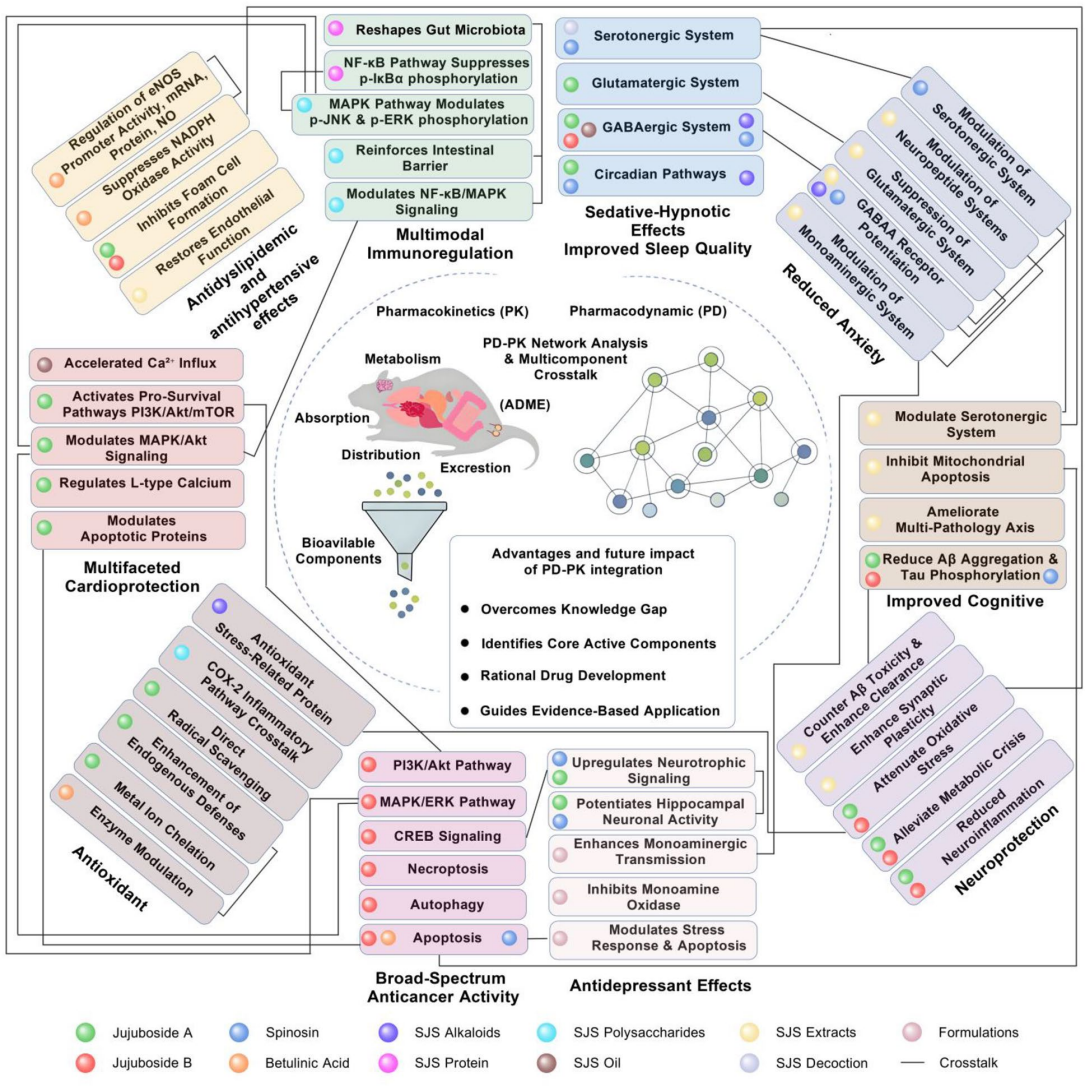
The landscape of multicomponent crosstalk in SJS deciphered by a PD‒PK network model. Created with BioGDP.com [22]

The therapeutic efficacy of SJS arises from a sophisticated multicomponent crosstalk, where diverse bioactive constituents—including saponins (e.g., JuA and JuB), flavonoids (e.g., spinosin), alkaloids (e.g., sanjoinine A and magnoflorine), fatty acids, and polysaccharides—interact synergistically across multiple pharmacological axes to produce integrated sedative-hypnotic, neuroprotective, anxiolytic, antidepressant, and antitumor effects. This polypharmacological profile is underpinned by a network of molecular interactions that modulate key neurotransmitter systems, signaling pathways, and metabolic processes. For instance, in the context of sleep regulation, jujubosides and their metabolites (e.g., jujubogenin) enhance GABAergic transmission via selective modulation of GABAA receptor subunits (α1, α5, and β2), while spinosin concurrently antagonizes 5-HT1A receptors to promote REM sleep and reduce sleep latency. Simultaneously, SJS oils—identified as the most potent hypnotic fraction—potentiate barbiturate-induced sleep without inducing tolerance, and alkaloids facilitate chloride influx through GABAA receptors, further amplifying neuronal inhibition. Beyond sedation, these components engage in cross-pathway modulation: jujubosides mitigate excitotoxicity by downregulating glutamate-mediated calcium influx and calmodulin activity, while flavonoids and alkaloids regulate circadian rhythms via the suprachiasmatic nucleus-pineal axis and serotonin/melatonin pathways. This multitarget engagement ensures broad-spectrum efficacy against diverse insomnia etiologies while maintaining physiological sleep architecture, reducing reliance on conventional sedatives (Fig. 8).

### Dose-dependent and pathway-convergent interactions of SJS components

The pharmacodynamic complexity of SJS is further enriched by pharmacokinetic interactions and metabolic transformations that dictate bioavailability, tissue distribution, and ultimate bioactivity. For example, JuB and jujubogenin—hydrolysis products of primary saponins—are identified as the actual absorbed metabolites responsible for GABAergic effects, highlighting the critical role of biotransformation in mediating therapeutic outcomes. Gut microbiota play a pivotal role in this process, as polysaccharides from SJS reshape microbial communities (e.g., increasing *Firmicutes* and *Verrucomicrobia*), which in turn enhance intestinal barrier integrity by upregulating occludin, P-gp, and MRP2 expression. This not only improves the absorption of bioactive compounds but also attenuates gut-derived inflammatory signaling, thereby indirectly modulating central nervous system functions via the microbiota–gut–brain axis. Moreover, component interactions exhibit dose-dependent synergy: low doses of flavonoids and saponins collectively enhance antioxidant defenses (e.g., upregulating SOD, CAT, and GPx), while JuA and betulinic acid coordinate to suppress oxidative stress and enhance endothelial NO synthase (eNOS) activity, conferring cardioprotection. In cancer models, JuB induces apoptosis through dual modulation of RIPK1/RIPK3/MLKL-mediated necroptosis and PI3K/Akt/MAPK inhibition, while synergizing with polyphenols to enhance chemosensitivity in colorectal carcinoma cells. These interactions are context-specific and often pathway-convergent; for instance, JuA’s antidepressant effects involve BDNF/TrkB/CREB upregulation, while its antiarrhythmic actions stem from L-type calcium channel modulation and Bcl-2/Bax rebalancing.

### Holistic botanical intervention: network pharmacology and therapeutic versatility of SJS

Ultimately, the integrated PD–PK network of SJS exemplifies a holistic botanical intervention strategy, where multicomponent synergy amplifies efficacy while minimizing off-target effects. The interplay between constituents—such as the potentiation of pentobarbital hypnosis by oils, saponins, and alkaloids—demonstrates how SJS achieves a balance between enhancement of inhibitory neurotransmission (GABAergic, serotonergic) and suppression of excitatory signals (glutamatergic, inflammatory). This network extends beyond neurological functions to encompass immunomodulation (via NF-κB/MAPK crosstalk), antioxidant defense (through radical scavenging and enzyme induction), and metabolic regulation (e.g., eNOS recoupling, lipid homeostasis). Importantly, the formulation-dependent optimization in traditional preparations (e.g., SJS–*Albizia julibrissin* pairs) further refines this network by leveraging herb-herb interactions to enhance bioavailability and target engagement. Thus, SJS serves as a paradigm for understanding how natural products achieve therapeutic versatility through multicomponent crosstalk, offering a template for developing multitarget therapies against complex diseases like insomnia, depression, neurodegeneration, and cancer.

## Conclusions

The use of TCM, particularly medicinal-food homology, for disease prevention and treatment has gained increasing popularity. SJS, known for its calming effects and ability to promote sleep with mild efficacy and minimal side effects, is widely favored. Its complex chemical composition leads to diverse medicinal effects. Phytochemical profiling has identified over 163 bioactive compounds in SJS, with saponins and flavonoids being the most predominant families [80, 134–140]. However, only a fraction of these compounds have been functionally characterized, and their precise sites of action remain unclear, as does the network of interactions between their various functions. Clinical validation of the therapeutic benefits and risk profiles of SJS is still pending large-cohort verification.

Pharmacokinetic studies of SJS components have mainly focused on JuA/B and spinosin, with limited exploration of the therapeutic implications of different tablet formulations, drug combinations, and a lack of in vivo human data. Current research on SJS bioactives prioritizes saponins, flavonoids, alkaloids, fatty acids, polysaccharides, vitamins, and amino acids, with no reports on other potential bioactive factors, such as peptides or small RNAs [141, 142].

Further investigation into the molecular targets and downstream signaling pathways of SJS using molecular and cell biology techniques is necessary to clarify its pharmacodynamic mechanisms. Modern analytical methods should be employed to assess the pharmacokinetic properties of bioactive constituents in vivo, providing a foundation for precise medication and personalized treatment. Additionally, the effects of different formulations and compatibility forms on pharmacokinetics should be studied to optimize the preparation process. Rigorous large-scale clinical trials are required to establish the risk–benefit balance of SJS in disease modification, providing robust evidence for its clinical application. Future research should integrate systems biology with large-scale clinical data to establish component–target–efficacy correlations, advancing the transition of traditional Chinese medicine to precision medicine.

## Data Availability

No datasets were generated or analysed during the current study.

## Abbreviations

Aβ_1–42_: β-Amyloid 1‒42
AC: Adenylate cyclase
AD: Alzheimer’s disease
ADMA: Asymmetric dimethylarginine
ADP: Adenosine diphosphate
ALT: Alanine aminotransferase
AMPAR: Alpha-amino-3-hydroxy-5-methyl-4-isoxazolepropionic acid receptor
AST: Aspartate aminotransferase
ATPase: Adenosine triphosphatase
Bax: Bcl-2-associated X protein
Bcl-2: B-cell lymphoma 2
BDNF: Brain-derived neurotrophic factor
CA1: *Cornu Ammonis*
CaM: Calmodulin
CeA: Central amygdala
*C*_max_: Maximum plasma concentrations
CNS: Central nervous system
COX: Cyclooxygenase
CRC: Colorectal carcinoma
CREB: CAMP-responsive element-binding protein
CRF/CRF1R: Corticotropin-releasing factor/corticotropin-releasing factor type 1 receptor
CRF-N/OFQ: Neuropeptide nociceptin/orphaninFQ
CTX: Cyclophosphamide
DDAH: Dimethylarginine dimethylaminohydrolase
DPPH: 1,1-Diphenyl-2-picrylhydrazyl
eNOS: Endothelial nitric oxide synthase
EPSP: Excitatory postsynaptic potential
ERK: Extracellular signal-regulated kinase
FasL: Fas ligand
GABA: γ-Aminobutyric acid
Glu: Glutamate
GluK1: Glutamate ionotropic receptor kainate type 1
GRP78: Glucose-regulated protein 78
5-HIAA: 5-Hydroxyindoleacetic acid
HPLC: High-performance liquid chromatography
5-HT1A: 5-Hydroxytryptamine-1A
HWE: Hot water extraction
ICa-L: L-type calcium current
I/R: Ischemia/reperfusion
ISO: Isoproterenol
JuA: Jujuboside A
JuB: Jujuboside B
JuA/B: Jujuboside A, and jujuboside B
LDL: Low-density lipoprotein
L-NAME: Nω-nitro-L-arginine methyl ester
MAPK: Mitogen-activated protein kinase
MDA: Malondialdehyde
MLKL: Mixed lineage kinase domain-like pseudokinase
MRP2: Multidrug resistance-associated protein 2
mTOR: Mammalian target of rapamycin
NADPH: Nicotinamide adenine dinucleotide phosphate
NMDA: N-methyl-D-aspartic acid
NREM: Non-rapid eye movement
NO: Nitric oxide
PAF: Platelet-activating factor
PAK: P21-activated kinase
PD‒PK: Pharmacodynamic‒pharmacokinetic
p-ERK: Phospho-extracellular signal-regulated kinase
PF-4: Platelet factor 4
P-gp: P-glycoprotein
PI3K/Akt: Phosphoinositide 3-kinase/protein kinase B
p-IκBα: Phospho-inhibitor of κB alpha
p-JNK: Phospho-c-Jun N-terminal kinase
REM: Rapid eye movement
RIPK1: Receptor-interacting protein kinase 1
ROS: Reactive oxygen species
RRLC: Rapid resolution liquid chromatography
SD: Sprague‒Dawley
SJS: Sour jujube seed
SJSD: SJS decoction
SJSP: SJS protein
SOD: Superoxide dismutase
T-AOC: Total antioxidant capacity
TGF-β1: Transforming growth factor-beta 1
Th: T helper cell
*T*_max_: Time to reach the maximum plasma concentration
TNBS: Trinitrobenzene sulfonic acid
TrκB: Tyrosine kinase receptor B
*t*_1/2_: Half-lives
UAE: Ultrasound-assisted extraction
UPLC-MS/MS: Ultrahigh performance liquid chromatography-tandem mass spectrometry
